# Mathematics anxiety: Effects of age, gender and culture

**DOI:** 10.1111/bjep.70057

**Published:** 2026-01-07

**Authors:** Ann Dowker

**Affiliations:** ^1^ Department of Experimental Psychology Oxford University Oxford UK

**Keywords:** affective processes, attitudes, cross‐cultural research, demographic factors ‐ gender, mathematical education, neuropsychology/neuroscience and education, stress and coping

## Abstract

**Background:**

Many studies have indicated that mathematics anxiety is a significant problem for many people and is an important topic for research. Mathematics anxiety is multidimensional. In particular, it is important to distinguish between worry and emotionality components, and between trait and state anxiety. Much research shows a reciprocal relationship between mathematics anxiety and performance adding to the importance of gaining a greater understanding of the factors involved in mathematics anxiety.

**Aims:**

This paper aims to review some studies of factors that have often been found to be associated with mathematics anxiety: age, gender, and culture, and to consider the evidence for these associations and the further research that should be done.

**Materials and Methods:**

The study involved reviewing a range of papers on the selected topics. In particular, the search terms ‘Age differences and mathematics anxiety’, ‘Gender and mathematics anxiety’ and ‘Culture and mathematics anxiety’ were put into Web of Science; and citations of relevant papers that emerged were also investigated.

**Results:**

Most studies suggest that mathematics anxiety increases with age, and that the relationship between mathematics performance and at least some aspects of mathematics anxiety increase with age. Most, though not all, studies, indicate that females experience more mathematics anxiety than males, and some suggest that there are gender differences in the relationship between mathematics anxiety and performance. Most studies show cultural differences in mathematics anxiety, while also suggesting that the relationship between mathematics anxiety and performance is fairly uniform across cultures.

**Discussion:**

Cross‐cultural studies in this area are. However, somewhat limited by the fact that culture is usually confounded with nationality. Moreover, there are studies of all these factors that give somewhat conflicting results.

**Conclusion:**

More research needs to be done in order to gain clearer answers, especially regarding the ways in which the effect of age, gender and culture interact with one another. The findings so far about the influences of age, gender and culture on mathematics anxiety have already had significant practical implications, but these need much further development.

Mathematical learning and performance are not only influenced by cognitive factors—both domain‐specific and domain‐general—but by attitudes and emotions. Such attitudes are multidimensional and include both positive and negative attitudes, with different researchers emphasising somewhat different attitudes. For example, Alken (1974) considered it important to assess both mathematics anxiety and two positive attitudes: enjoyment of mathematics and value placed on mathematics. Fennema and Sherman (1976) developed scales to measure several different attitudes to mathematics: attitudes toward success in mathematics; perceiving mathematics as a male domain; mother's and father's support in mathematics; teachers' support in mathematics; confidence; mathematics anxiety; effectance motivation (seeking vs. avoiding challenge) and perceived usefulness of mathematics. Levine and Pantoja (2021) focussed on four attitudes: math anxiety, math self‐concept, mindset (growth vs. fixed) and math‐gender stereotype. Across different research groups, time periods and categorisation systems, mathematics anxiety is one of the most frequently and consistently mentioned.

There are several reasons why mathematics anxiety should be at the forefront of research on attitudes to mathematics. It tends to correlate positively with other negative attitudes to mathematics; negatively with positive attitudes; and, as will be discussed, negatively with mathematical performance. It is painful and distressing, especially if experienced frequently and/or to a marked degree. And high levels of mathematics anxiety are quite frequent in the population (Ashcraft, 2002; Dowker et al., 2016; Maloney & Beilock, 2012).

Mathematics anxiety is defined as “a feeling of tension and anxiety that interferes with the manipulation of numbers and the solving of mathematical problems in…ordinary life and academic situations” (Richardson & Suinn, 1972, p. 551). People have been expressing mathematics anxiety for centuries. A verse dating back at least to the 16th century states that: “Multiplication is vexation…And practice drives me mad.” It is a sufficiently serious problem for many people that a Maths Anxiety Trust was recently set up in the UK (https://mathsanxietytrust.com). Studies differ as to the exact frequency of mathematics anxiety, presumably depending on the exact nature of the sample and the exact criteria used for diagnosing mathematics anxiety; but most suggest a worryingly high proportion. For example, Richardson and Suinn (1972) estimated that 11% of university students show high enough levels of mathematics anxiety to be in need of counselling. Ashcraft and Moore (2009) estimated that 17% of the population have high levels of mathematics anxiety. Johnston‐Wilder et al. (2014) also found that about 30% of a group of apprentices showed high mathematics anxiety, with a further 18% affected to a lesser degree.

Mathematics anxiety is not only common in the population post‐school, but importantly, among school pupils, who are in the process of learning mathematics. Though Chinn (2009) found high mathematics anxiety in a relatively low – though still significant – proportion (2–6%) of secondary pupils, many studies have found a higher proportion. For example, Carey et al. (2019) found that 11% of a large sample of secondary school students in south east England exhibited high mathematics anxiety. While many researchers find lower mathematics anxiety in primary school children, Krinzinger et al. (2009) found that 32.8% of a group of German second‐and third‐grade pupils reported high or very high mathematics anxiety.

From a research perspective, the construct has been an important topic of study at least since the concept of ‘number anxiety’ was introduced by Dreger and Aiken (1957). It has received increasing attention in recent years, in conjunction with the generally increased focus in many countries on mathematical performance. It is often discussed in relation to other attitudes to mathematics, such as enjoyment, self‐efficacy, evaluation of mathematics as useful, etc. There is some debate as to whether mathematics anxiety should be classified as an attitude at all, as anxiety is predominantly emotional rather than evaluative. We here, however, follow some previous researchers, such as Ma and Kishor (1997) in treating mathematics anxiety as an attitude, especially as some attitude theories, such as Breckler's (1984) ABC theory, consider that attitudes have both cognitive and affective components.

## HOW IS MATHEMATICS ANXIETY MEASURED, AND WHAT DO WE MEASURE?

Most commonly, mathematics anxiety is measured through self‐report, usually in questionnaires, where participants are asked to rate their own level of anxiety in a variety of mathematics‐related situations. Such self‐report measures usually show good reliability (Cipora et al., 2019), though studies also suggest that internal consistency can never be perfect, as mathematics anxiety is not a unitary construct. Anxiety may differ between different types of mathematics‐related situations, e.g. learning mathematics, taking mathematics tests, doing mathematics in everyday practical situations, and simply being exposed to numerical stimuli. It is also likely that individuals may experience different forms and degrees of anxiety for different types of mathematical content: whole number arithmetic; fractions and decimals; word problems; algebra; geometry; etc. There has been less research on differences in anxiety for different forms of mathematical content than on differences in anxiety for different mathematics‐related situations. However, several studies indicate that mathematics anxiety as typically defined and statistics anxiety can dissociate.

One key issue in the study and measurement of mathematics anxiety is the distinction between state and trait anxiety, first formulated by Spielberger (1972). The former refers to a person's current emotional state; the latter to a long‐term disposition to experience that emotional state. While a person with high mathematics anxiety as a trait is likely to experience state mathematics anxiety frequently, it is not an inevitable consequence, as some people with high trait mathematics anxiety may avoid state anxiety by successfully avoiding mathematics‐related activities altogether. On the other hand, people with relatively low trait mathematics anxiety may still experience episodes of high state anxiety. This may relate to the situation and content effects discussed above, and, for example, a person who is not usually anxious about mathematics may become so when taking a high‐stakes examination in the subject. More random‐seeming variations in mood or in the details of how mathematics anxiety is assessed may also be influential. In order to investigate whether an individual's self‐reported mathematics anxiety on a given occasion represents a trait or a short‐term state, it would be ideal to test them repeatedly.

While, if the componential nature of mathematics anxiety is taken into account, self‐report measures are generally seen to be reliable, are they also valid? They do seem to have significant criterion validity in important ways: most studies, as indicated above, show a significant relationship between mathematics anxiety and mathematics performance, and mathematics anxiety is negatively associated with choice to study mathematics beyond compulsory age, or to pursue STEM‐related careers (Brown et al., 2010; Chipman et al., 1992; Ferdinand et al., 2024; Levy et al., 2021). One caveat is that we cannot be sure to what extent individuals are explicitly aware of their own levels of mathematics anxiety and this may be particularly problematic in studies involving young children. Moreover, responses may be influenced by social desirability biases. Participants may emphasise or exaggerate their levels of mathematics anxiety, because they think that it will appear boastful to express too much confidence in their mathematical skills, or because they think that it is ‘normal’ to fear and dislike mathematics. Alternatively, they may underestimate their levels of mathematics anxiety because they think that admitting such anxiety reflects on their mathematical competence, or because they think that expressing anxiety is weak. The issue of social desirability biases is potentially particularly important when comparing different genders or cultural groups, who may differ in what they consider socially desirable.

The main alternative to self‐report measures is physiological measures of anxiety, such as heart rate, cortisol levels, skin conductance and brain imaging in response to numerical stimuli and mathematical tasks. These could be seen as having greater objectivity and less risk of bias than self‐report measures. However, they are more expensive and practically challenging to apply, and therefore are less used and may result in smaller samples even when they are used. Moreover, it is not entirely clear that what they are measuring is mathematics anxiety as a trait. Heart rate and skin conductance have not been consistently found to correlate strongly with questionnaire measures of mathematics anxiety (Hopko et al., 2005; but see Hunt et al., 2017 for some positive findings about correlations with heart rate and blood pressure). This could reflect a problem with the questionnaire measures, but could also indicate that these physiological measures as used thus far are too crude to give reliable results, or that they are measuring something else, such as arousal, rather than anxiety. Salivary cortisol levels tend to show greater correlation with questionnaire measures (e.g. Mattarella‐Micke et al., 2011; Pletzer et al., 2010) but the correlation is not perfect. Both ERP and fMRI methods of brain imaging suggest some correlation between mathematics anxiety and activation related to cognitive control and emotional regulation (Avancini & Szucs, 2019), but the complexity of activation patterns and number of brain areas involved make interpretation complicated. With regard to all physiological measures, individual differences in physiological reactivity may play a role and much more research is needed on the topic.

Moreover, physiological measures in general could be seen as measures of state anxiety (level of mathematics anxiety at a specific time) rather than trait anxiety (general disposition toward mathematics anxiety). The difficulty in drawing conclusions about trait mathematics anxiety from physiological measures is increased by the fact that they are more difficult than self‐report measures to repeat on multiple occasions to check for consistency.

## THE MULTIDIMENSIONAL NATURE OF MATHEMATICS ANXIETY

Mathematics anxiety is multidimensional. Anxiety scales often include different dimensions: e.g. anxiety about numbers as such, anxiety about learning and studying mathematics, anxiety about mathematics tests. In particular, a number of studies (e.g. Sorvo et al., 2017; Wigfield & Meece, 1988) suggest a distinction between a ‘worry component of mathematics anxiety (mainly involving performance anxiety) and an ‘emotionality’ component (involving a fearful reaction to mathematical stimuli).

It is also important to note the distinction between trait mathematics anxiety and state mathematics anxiety (Lievore et al., 2024; Mammarella et al., 2023). Trait mathematics anxiety refers to a consistent tendency toward anxiety about some or all aspects of mathematics, while state mathematics anxiety refers to anxiety at a specific time. Trait anxiety is likely to incorporate multiple instances of state anxiety, but state anxiety might be specific to an isolated occasion, e.g. just before an important examination.

## IS MATHEMATICS ANXIETY A SEPARATE SPECIFIC ENTITY?

All of this gives rise to the question of how specific mathematics anxiety is. Should it indeed be seen as a specific entity, or is it just one aspect of general anxiety or academic test/examination anxiety?

Mathematics anxiety has indeed been found to correlate significantly with measures of general anxiety. Hembree (1990) found a mean correlation of .35 between the Mathematics Rating Scale (MARS) and a measure of general anxiety. Wang et al. (2014) obtained evidence that genetically based differences in general anxiety contribute to genetic differences in mathematics anxiety.

It correlates even more with test anxiety. Several studies suggest that mathematics anxiety is more closely related to test anxiety than to measures of academic ability and performance (Ashcraft et al., 1998; Hembree, 1990). Such studies typically show correlations from .3 to .5 between measures of mathematics anxiety and test anxiety. However, mathematics anxiety cannot be reduced to either test anxiety or general anxiety. Mathematics anxiety does exist as a specific entity. This is shown by the fact that different measures of mathematics anxiety correlate more highly with one another (.5–.8) than with test anxiety or general anxiety (Ashcraft & Ridley, 2005; Dew et al., 1984; Hembree, 1990).

## RELATIONSHIPS BETWEEN MATHEMATICS ANXIETY AND PERFORMANCE

One of the most consistent findings about mathematics anxiety is that it is negatively associated with mathematics performance (Abín et al., 2020; Barroso et al., 2021; Carey et al., 2016; Caviola et al., 2022; Hembree, 1990; Ma & Xu, 2004; Namkung et al., 2019). This link is found in virtually all countries, where it has been studied (Foley et al., 2017).

The negative correlation between mathematics anxiety and mathematics performance has sometimes been explained purely on the grounds that anxiety impedes performance. This is sometimes termed the Deleterious Anxiety theory. Certainly, anxiety is likely to have a deleterious effect on performance. One reason is that anxiety is likely to deter people from engaging with and practicing mathematics beyond what is strictly compulsory and therefore will restrict their learning opportunities. Another is that, when a person performs mathematical tasks, mathematics anxiety is likely to add to the load on working memory, and thus to reduce the cognitive resources available for solving the mathematical problems. Several studies do indicate that mathematics anxiety interferes with mathematical problem solving by disrupting working memory resources (Krinzinger et al., 2009; Skagerlund et al., 2019). Cargnelutti et al. (2017) studied children longitudinally from second to third grade and found that mathematics anxiety predicted future mathematical attainment much more than attainment predicted future mathematics anxiety. Moreover, performance tends to improve if mathematics state anxiety is reduced (Park et al., 2014), and there is less of an association between anxiety and performance if mathematics tasks are carried out without time pressure (Faust et al., 1996).

Alternatively, it is sometimes suggested that the direction of causation is the reverse: that mathematical weaknesses lead to anxiety. This is often known as the Deficit Theory and argues that mathematical deficits will result in negative experiences of failure and confusion in mathematical tasks, which will lead to anxiety. For example, Maloney et al. (2011) have suggested that mathematics anxiety may originate from deficits in basic numerical processing, which lead to mathematical difficulties, which lead to anxiety Support for this theory comes from some longitudinal studies (Ma & Xu, 2004; Wang et al., 2020), which have shown that mathematics scores predict subsequent levels of mathematics anxiety, However, it is quite possible to have mathematics anxiety despite average or above‐average mathematical attainment, and to have low attainment or even dyscalculia; without mathematics anxiety. (Devine et al., 2018). Mathematics anxiety cannot be solely blamed on mathematical deficits.

At present, the Reciprocal Theory seems to be most justified by the evidence. This is the theory that there are links in both directions. Several longitudinal studies of pupils in various age groups and various countries have indicated that mathematics anxiety predicts subsequent mathematical attainment *and* that mathematical attainment predicts subsequent mathematics anxiety (Aldrup et al., 2020; Du et al., 2021; Gunderson et al., 2018; Luo et al., 2014; Pekrun et al., 2017). Szczygieł et al. (2024) found such a reciprocal relationship as early as first and second grade. Mathematics anxiety impairs performance, and poor performance increases anxiety. This can easily become a vicious circle (Jameson, 2014).

In studying group differences (e.g. age‐group, gender or national difference) mathematics anxiety, it is important to consider two possible types of difference: differences in levels of mathematics anxiety itself and differences in the relationship between mathematics anxiety and performance. These will be discussed separately in each of the sections on possible group differences.

When considering differences between studies regarding relationships between mathematical performance, mathematics anxiety and other factors, it is important to remember that methodological differences between studies can contribute to the differences. Statistical methods are not the focus of the present paper, but it should be noted that, for example, results may differ according to whether a correlational analysis or a regression model is used. Correlations often change strength or even cease to be significant when several variables are included in the same regression model.

For example, some studies suggest that factors such as self‐concept and engagement may partially or completely mediate the relationship between anxiety and performance, at least in some groups. Van der Beek et al. (2017) studied pupils in eight secondary schools in the Netherlands and found that relationships between mathematics performance and both mathematics anxiety and mathematics enjoyment ceased to be significant after controlling for mathematics self‐concept. Pei et al. (2025), in a study involving pupils in three Chinese secondary schools found that the relationship between mathematics anxiety and performance disappeared after controlling for mathematical engagement and suggested that mathematical engagement may mediate the mathematics anxiety‐performance relationship.

## GROUP DIFFERENCES IN MATHEMATICS ANXIETY

As indicated above, a lot of research has been done on relationships between mathematics anxiety and individual characteristics such as mathematics attainment and general and test anxiety. There has also been significant focus on potentially important group differences that may affect mathematics anxiety. In particular, factors that are often proposed to be associated with mathematics anxiety include age, gender and national/cultural differences. Studying such factors is of interest both in its own right, and because it may indicate which groups are likely to be particularly vulnerable to the condition and should be a focus for strategies to prevent or ameliorate it. These factors will now be discussed in turn.

## AGE DIFFERENCES IN MATHEMATICS ANXIETY

Most studies of mathematics anxiety have been carried out with adolescents and adults, though research on mathematics anxiety in younger children has increased recently (Cargnelutti et al., 2017; Dowker et al., 2019; Gattas, 2024; Jameson, 2014; Petronzi et al., 2019; Szczygiel, 2020a, 2020b; Szczygieł & Pieronkiewicz, 2021; Vukovic et al., 2013).

Most studies that include younger children suggest that primary school children's attitudes to mathematics tend to be positive. Most studies also suggest that mathematics anxiety and other negative attitudes and emotions regarding mathematics increase with age, during the primary school years and especially during the secondary school years (e.g., Batchelor et al., 2019; Gierl & Bisanz, 1995; Ma & Kishor, 1997; Sumpter & Sollerman, 2023; Wigfield & Meece, 1988).

These findings are important both practically and for their implications for the concept of mathematics anxiety. They are of practical importance because they indicate a serious problem for mathematics education: the frequent deterioration of attitudes to mathematics with age and educational stage. They are of theoretical importance because of the mostly not fully answered questions that they bring about the nature and causes of mathematics anxiety. Why does mathematics anxiety appear to increase with age? Is it something to do with developmental changes as such, perhaps reflecting greater capacity for self‐evaluation and self‐criticism? Or is it due to greater experience of failure and frustration in the school context? Or is it due, not to age changes as such, but to changes in the type of mathematics to which children are exposed as they progress through school? For example, secondary school pupils may have to deal with more abstract types of mathematics than primary school children, and more abstract mathematics may elicit more anxiety.

However, the fact that mathematics anxiety is usually found to be greater in older children does not mean that younger children are immune to it. Studies do show that mathematics anxiety can be seen in some children in the early years of primary school (Jameson, 2014; Krinzinger et al., 2009; Ma & Kishor, 1997; Ramirez et al., 2013; Szczygiel, 2020a, 2020b; Vukovic et al., 2013). Petronzi et al. (2019) found significant mathematics anxiety in children as young as five. While most studies show increased anxiety with age, Sorvo et al. (2017) found that Finnish children in Year 2 showed greater anxiety in Year 2 than children in later elementary grades. The fact that different studies give somewhat different results as regards the frequency of mathematics anxiety in younger children may be due to differences in sampling and the levels of academic pressure and testing to which the children have been subjected and/or to differences in the measures used to assess mathematics anxiety. These issues will be discussed in more detail with regard to conflicting results about the relationship between anxiety and performance in young children, where findings differ more strikingly.

At the other end of the age scale, most studies of mathematics anxiety have looked at people within the education system: schoolchildren; university students; occasionally apprentices. This means that the influence of test/course anxiety is likely to be prevalent and may affect responses to measures of other forms of anxiety. It also means that samples are likely to be relatively young. One of relatively few studies to involve adults not currently in education was that of Hart and Ganley (2019), who studied 1000 adults, who were not currently undergoing formal education. Mathematics anxiety was approximately normally distributed, Findings about its correlates were similar to those obtained in studies of younger participants. Mathematics anxiety correlated positively with general anxiety and test anxiety and negatively correlated with measures or mathematical anxiety and knowledge. It tended to be lower in people with higher levels of education, and in people who were pursuing STEM‐related careers.

## AGE DIFFERENCES IN RELATIONSHIPS BETWEEN MATHEMATICS ANXIETY AND PERFORMANCE

Most studies suggest levels of mathematics anxiety change, and usually increase, with age. Does the relationship between mathematics anxiety and performance also change with age, or does this remain constant?

Some studies do suggest that relationships between attitudes and performance can change with age. A meta‐analysis by Ma and Kishor (1997) indicated that the relationship between mathematics anxiety and performance increases with age. However, the evidence is somewhat conflicting, for reasons that will be discussed in the next section.

Dowker et al. (2012) obtained results which suggest that performance in primary school children is not related to mathematics anxiety in the ways commonly found in studies of older participant. This study suggested that it is instead related to self‐rating in mathematics. Self‐rating refers to individuals' assessment of their own mathematical competence. There are several related constructs, notably identification of oneself with mathematics and mathematical self‐efficacy (confidence in one's ability to take steps to solve new problems, learn new material and improve future mathematical performance). In this case, self‐rating refers more specifically to assessment of one's current level of ability in aspects of mathematics.

Dowker et al. (2012) looked at the relationships between primary school children's attitudes to mathematics and their actual performance. In this study, 44 Year 3 children (7–8 years old) and 45 Year 5 children (9–10 years old) from English primary schools were given the British Abilities Scales Basic Number Skills subtest. They were also given the Mathematics Attitude and Anxiety Questionnaire (Thomas & Dowker, 2000).

They rated seven school mathematical activities (e.g., written sums; mental maths; understanding the teacher) on four 5‐point pictorial scales:

1. How much they liked the activity:
2. How good they thought they were at the activity;
3. How unhappy they would be if they couldn't do it;
4. How anxious they would feel if they couldn't do it

Children's self‐rating of competence appeared quite high, with a mean score of 27.66 out of a possible 35. Their enjoyment of mathematics was lower, but also positive (mean 24.76). Anxiety and unhappiness at failure were somewhat lower and were significantly correlated, though not identical.

Self‐rating correlated significantly with (lack of) anxiety and (lack of) unhappiness. Actual mathematics score correlated significantly with self‐rating, but not with any other attitude measure. Regression analyses showed that in Year 5, self‐rating was the only attitude that independently predicted mathematics score, while in Year 3, no attitude was an independent predictor. Some other studies have given similar results for primary school children suggesting that among young children, performance is not significantly related to anxiety (Cain‐Caston, 1993; Krinzinger et al., 2009; Wood et al., 2012) but is more related to self‐rating of mathematical ability.

Looking at still younger children, Dowker et al. (2019) studied 67 English and 49 Chinese children at the end of their first year of school. The participants were given the same tests as in the study by Dowker et al. (2012): the Mathematics Attitude Questionnaire and the British Abilities Scales Basic Number Skills test. Attitudes were similar to those for the older primary school children in the previous study. The Chinese children performed better in the arithmetic test and also rated themselves higher than the English children but did not differ in other attitudes. There were few gender differences.

Mathematics anxiety was not associated with performance in either group. The only relationship that was found between attitudes and performance was that self‐rating in mathematics was correlated with better performance in the English group. A regression analysis showed that both self‐rating and unhappiness at failure (but not anxiety) were independent predictors of performance in the English group. There were no significant relationships between attitudes and performance in the Chinese group.

Dowker et al.'s (2012, 2019) conclusion was that younger children's mathematical performance correlates mainly with their self‐rating of their mathematical ability, and that the negative correlation between mathematical ability and performance only becomes evident at a somewhat later age. A subsequent study (Dowker et al., 2022, 2023) puts this conclusion somewhat into question.

The latter study investigated children's and adolescents' attitudes to mathematics, with a particular focus on whether and how these are affected by age and gender. 216 pupils from Years 2 (6‐to 7‐year‐olds), 6 (10‐to 11‐year‐olds), 9 (13‐to 14‐year‐olds) and 12 (16‐to 17‐year‐olds) participated in the study. They were equally divided as to year group, and each year group was approximately equally divided by gender. The same measures of attitudes and performance were used as in the earlier studies by Dowker et al. (2012) and Dowker et al. (2019).

As predicted, mathematics anxiety increased with age, with a marked difference between primary and secondary age pupils. Other attitudes also declined with age. The study supported the hypothesis that attitudes to mathematics decline with age. Older groups liked mathematics less, and rated themselves significantly lower than younger groups. Unhappiness at failure increased with age, with a marked difference between Year 2 and older groups. All these age differences remained significant after controlling for Basic Number Skills (BNS) standard score.

Correlation analyses indicated that none of the attitude variables correlated with BNS score in Year 2, but that self‐rating and liking for maths did so in Year 6 and all did so in Years 9 and 12, However, most of these relationships ceased to be significant when all variables were included together in multiple regressions. However, most of these relationships ceased to be significant when all variables were included together in multiple regressions. Multiple regressions were carried out for each gender and each year group separately, and for the group as a whole, with BNS standard score as the dependent variable, and the attitude scores as predictors. For the whole group, the four predictors explained 34% of the variance in BNS standard score. Self‐rating was a significant predictor, but no other predictor was significant. Results were similar for each year group separately, and for each gender separately: self‐rating was the only significant independent predictor of performance. The exception was Year 2: no attitude variable was a significant predictor of performance for these youngest children. Thus, in this study, self‐rating rather than anxiety was the main independent predictor of performance, among older as well as younger participants.

By contrast, some studies have shown that mathematics anxiety correlated with performance not only in older children, but even in the early years of elementary school (Dossey et al., 1988; Newstead, 1998; Ramirez et al., 2013; Vukovic et al., 2013; Wu et al., 2012). Svraka et al. (2024) found such a relationship between general anxiety and mathematics performance even in a group of Hungarian kindergarten children, with a stronger relationship for girls than for boys. The authors suggested that high general anxiety might be an early precursor to high mathematics anxiety, though mathematics anxiety and attitudes were not tested in this study.

## AGE DIFFERENCES: SOME REASONS FOR CONFLICTING RESULTS

Why do we have these conflicting results? There seem to be three major possibilities: (1) cultural and other sampling differences; (2) the type of mathematics anxiety being tested; and (3) the role of working memory.

1. Samples have been somewhat different in different studies: involving children from different national backgrounds. The studies cited above, that. showed a relationship between mathematics anxiety and performance even in young children, were mostly carried in the USA, with the exception of Svraka et al.'s study, which involved Hungarian children. It is therefore possible that there are cultural and/or educational factors, which lead to an earlier development of the anxiety‐performance relationship in the USA than in the UK and some other countries. However, we do not know at present what these factors might be. The likelihood of this explanation is, moreover, somewhat called into question by the fact that the curriculum in the USA is decided predominantly at school district level, so that educational practices are not uniform across the nation.
2. Differences between studies may reflect the type of mathematics anxiety being investigated: in particular, the distinction between ‘worry’ (performance anxiety) and ‘emotionality’ (fear and anxiety elicited by the very presence of mathematical stimuli). Wigfield and Meece (1988) found that emotionality had the stronger negative correlation to performance, and the correlation was found even in elementary school children, whereas worry only showed a relationship to performance when children reached adolescence. Some studies have mainly assessed the emotionality component of mathematics anxiety, for example, those that used measures based on Richardson and Suinn's (1972) Mathematics Rating Scale (MARS) or MARS‐Elementary (Suinn et al., 1988). Such studies have tended to find such a relationship even in elementary school children (Vukovic et al., 2013; Wu et al., 2012). Studies that used measures that emphasised performance anxiety, such as the Mathematics Attitude and Anxiety Questionnaire (MAQ) developed by Thomas and Dowker (2000) have tended not to show such a relationship in younger children (Dowker et al., 2012; Krinzinger et al., 2009; Wood et al., 2012). The few studies that have studied both forms of mathematics anxiety in young children have suggested that younger children do show a relationship between mathematics performance and emotionality, but not between mathematics performance and worry/performance anxiety (Harari et al., 2013; Sorvo et al., 2017).
3. Another possible reasons for changes in the relationships between mathematics anxiety and performance may involve relationships between working memory, anxiety and mathematics achievement. Vukovic et al. (2013) followed 113 children, from second to third grade. Mathematics anxiety showed a negative relationship with mathematical progress from second to third grade for children with *higher* levels of working memory than for those with lower levels of working memory. This may seem counter‐intuitive in view of the evidence that mathematics anxiety affects arithmetical performance by overloading working memory. It might initially have been predicted that this effect might have been most damaging in children, who already had lower levels of working memory to start with. However, we may here be talking about children who are at an educational stage of moving from predominantly counting‐based strategies to strategies based more on mental calculation, which place greater demands on working memory. Children with higher levels of working memory may make greater use of the latter type of strategy, and their performance may therefore be more disrupted by the effects of mathematics anxiety on working memory. By contrast, Johnson and Gronlund (2009) found the reverse relationship in a group of adult undergraduates. The relationship between mathematics anxiety and mathematics performance was stronger in participants with lower working memory levels than those with higher working memory levels. This may be due to all participants relying on mental calculation strategies, which may have been more easily disrupted by the load placed by anxiety on working memory in individuals, who already had working memory limitations.

So far, we have been looking at age differences in the relationship between mathematics anxiety and performance mainly in terms of how anxiety might affect performance at different ages. However, in view of the reciprocal relationship between anxiety and performance, there may also be age differences in how performance affects anxiety. As pupils have increasing exposure to success and failure in mathematics tasks in the classroom and in examinations, low performance levels may be associated with increased anxiety by causing repeated experiences of failure, while high performance levels may be associated with reduced anxiety by causing repeated experiences of success.

It is also important to take into account possible interactions between mathematics anxiety and other attitudes to mathematics. Mathematics anxiety is not invariably associated with low motivation toward mathematics, and there is some evidence that mathematics anxiety is not as strongly associated with reduced performance in individuals who have high mathematics motivation (Wang et al., 2015, 2018).

Taken together, studies suggest that mathematics anxiety increases with age during the school years. This may be due to greater self‐criticism, more exposure to academic pressure, experiences of failure, changes in the content of the mathematics curriculum, or any combination of these. Some studies also suggest that the relationship between mathematics anxiety and mathematical performance increases with age, though the evidence here is a little more conflicting for reasons that have been discussed above.

There are a number of limitations in current research. Most studies have not compared different age groups systematically on a wide variety of anxiety measures. They have not included in their age comparisons measures of other cognitive abilities that may be associated with mathematics (working memory, executive functions, language, spatial ability) or with anxiety (general anxiety, test anxiety). And they have very rarely attempted to distinguish between the effects of age differences as such and the effects of differences in the mathematical topics being taught at different ages. Clearly, there is much more research to be done in this area.

## GENDER DIFFERENCES IN MATHEMATICS ANXIETY

Another factor that is often found to influence mathematics anxiety is gender. For example, the PISA international comparisons have consistently found greater mathematic anxiety in girls than boys in most countries (OECD, 2014, 2023). This is not the result of gender differences in actual mathematical performance. Most studies nowadays indicate that, where girls and boys have similar educational opportunities in mathematics, they perform similarly in mathematics, though males are more likely to choose to take post‐compulsory courses in education and to pursue STEM careers (Levine & Pantoja, 2021). This was already found in the early 21st century (Spelke, 2005) and is generally supported by the data from recent international assessments. An analysis by Thomson et al. (2020) of the data from the TIMSS 2019 international comparisons showed that in Year 4, girls and boys performed equally well in 47% of the countries tested, while boys performed better in 47% of the countries, and girls performed better in just under 7%. By contrast, in Year 8, girls and boys performed equally well in 67% of the countries tested and the rest were almost equally divided between those where boys did better and those where girls did better.

Most studies have suggested that any gender differences in mathematics attainment only begin in the secondary years and are not found among primary school children (e.g. Hyde et al., 2008; Kersey et al., 2018, 2019; Spelke, 2005). Therefore, any gender differences in attitudes and emotions regarding mathematics cannot be attributed to differences in performance or attainment. However, it is generally found that females have more negative attitudes to mathematics, rate themselves lower in mathematics, and are more anxious about mathematics (Callan, 2015; Devine et al., 2012; Else‐Quest et al., 2010; Hembree, 1990; Kyttala & Bjorn, 2010; Wigfield & Meece, 1988; Williams et al., 2024).

Before investigating the reasons for gender differences in mathematics anxiety, it must be pointed out that not all studies do show such gender differences. The Mathematics Anxiety Trust (2018) carried out a survey of 2000 British secondary school students and found little or no difference between males and females in mathematics anxiety. Dowker, Cheriton and Horton's study (Dowker et al., 2022, 2023), described above with regard to age differences in attitudes to mathematics, also looked at gender differences. It partially supported the hypothesis that boys would have more positive attitudes to mathematics than girls but did not show gender differences in mathematics anxiety. Boys and girls scored similarly on actual mathematics performance, but boys liked mathematics significantly more and rated themselves significantly higher in mathematics than girls. For self‐rating, there was a significant interaction between Year Group and Gender. In Year 2, girls rated themselves slightly higher than boys did; but their self‐rating declined more rapidly thereafter. There were, however, no gender differences in mathematics anxiety or in unhappiness at failure.

However, the majority of studies do show females to be more maths anxious, though the differences are often not very large, and effect sizes tend to be quite small. There are several factors that may contribute to the differences that are found:

## POSSIBLE REASONS FOR GENDER DIFFERENCES IN MATHEMATICS ANXIE

1. Females tend to be more anxious about many things. Most studies indicate that females score higher on general anxiety measures and on the closely related personality trait of Neuroticism than males (e.g., Chapman et al., 2007; Costa et al., 2001; Feingold, 1994) and show higher prevalence of clinical anxiety disorders (McLean et al., 2011). Similarly, test anxiety is higher in females than males (e.g., Devine et al., 2012; Putwain & Daly, 2014) and, as stated above, correlates significantly with mathematics anxiety. However, these factors cannot fully explain gender differences in mathematics anxiety, as the latter are generally found to persist even after controlling for general anxiety and test anxiety (e.g.Devine et al., 2012; Rossi et al., 2023).

However, it is possible that the extent to which general anxiety mediates the relationship between gender and mathematics anxiety depends on age. Szczygiel (2020a) found that general anxiety completely mediated the relation between gender and maths anxiety in first and second grade children, in contrast with the findings with regard to older children (Devine et al., 2012; Rossi et al., 2023). Thus, it may be that gender differences in mathematics anxiety are initially a function of general anxiety. Gender differences in specific mathematics anxiety only emerge as a result of later experiences of test and evaluation pressures, criticism from teachers and peers, confusion in mathematical tasks and negative self‐evaluation and negative cultural attitudes to mathematics, to all of which girls may be more vulnerable as a result of their greater general anxiety. However, more research is needed before we can draw firm conclusions as to whether specific gender differences in mathematics anxiety are consistent across the age range, or only develop as children get older.

2 Parents and teachers may experience mathematics anxiety themselves, and may transmit greater anxiety to girls than to boys. Beilock et al. (2010) found that mathematics anxiety in female elementary school teachers was associated with increased mathematics anxiety in their female, but not their male, pupils. It would be interesting to investigate whether there is a similar specific transmission of mathematics anxiety from male elementary school teachers to boys, but this would be difficult to study due to the relatively small number of male elementary school teachers.

If parental mathematics anxiety does influence their children's mathematics anxiety, this might provide some explanation of why mathematics anxiety tends to be greater in girls, as mothers are likely to have more mathematics anxiety than fathers and might be more likely to transmit it to children of the same gender. This is somewhat speculative, however, especially as studies of effects of parental mathematics anxiety often focus mainly on mothers and do not compare the effects of fathers' and mothers' mathematics anxiety. Vanbinst et al. (2020) found that children's mathematics anxiety was correlated with their mothers' but not their fathers' mathematics anxiety, but this did not differ for boys and girls. However, the effect was not very strong even for mothers' mathematics anxiety. On the other hand, Szczygiel (2020b) found that mathematics anxiety in fathers, but not mothers or teachers, was associated with math anxiety in first‐grade children of both sexes and third‐grade girls.

3 One factor that may contribute to gender differences in mathematics anxiety is the existence of gender stereotypes. Gender stereotypes are preconceived, generalise and usually pervasive ideas about traits associated with different genders: in this case, concerning alleged gender differences in mathematical ability Traditionally, males have been regarded as better than females at mathematics. Beilock et al. (2010) found that the endorsement of this gender stereotype had some effect on children's susceptibility to intergenerational teacher‐pupil transmission of mathematics anxiety. They found that students' gender stereotype beliefs about mathematics mediated the relationship between female teachers' mathematics anxiety and female students' mathematics achievement Some research suggests that gender stereotypes may negatively affect females' mathematics performance specifically through stereotype threat. If females (but not males) are reminded of the stereotype that males are better than females at mathematics, they tend to perform worse in a mathematics test than if they are given the same test with no such reminder (Beilock et al., 2007; Flore & Wicherts, 2015; Spencer et al., 1999). Some studies suggest this to be the case even for primary school children (Ambady et al., 2001; Galdi et al., 2014; Huguet & Régner, 2009). However, the effects of stereotype threat on performance are usually not found to be very large (Flore & Wicherts, 2015); not all researchers have found them at all; and most studies have not looked at mediating effects of mathematics state or trait anxiety. Thus, the effects of stereotype threat have been studied much more from the point of view of performance than anxiety, and, while the assumption tends to be that anxiety plays a mediating role, this has rarely been studied directly.

However, some recent studies have indicated that gender stereotypes do affect mathematics anxiety; that they have different effects on mathematics anxiety in men and women, and, more surprisingly, that this is not purely a matter of their affecting women negatively and men positively. Rossi et al. (2022) found that men who endorsed mathematics gender stereotypes reported a somewhat higher mathematical self‐concept than those who did not, but also reported somewhat higher numerical anxiety (but not test anxiety). However, in the case of men, effects of test and numerical anxiety on performance were entirely mediated by self‐concept; there was no additional independent influence of anxiety on performance. In the case of women, the effects of gender stereotype endorsement were more unambiguously negative: analysis indicated that it was associated with both increased test anxiety and increased numerical anxiety which seemed to lead to reduced self‐concept which was in turn associated with reduced performance. There also appeared to be a direct negative effect of numerical anxiety on women's performance, separately from its effect on self‐concept

4 It must be remembered that females reporting greater mathematics anxiety does not necessarily mean objectively greater mathematics anxiety (Flessati & Jamieson, 1991). It might be more socially acceptable for females than males to express mathematics anxiety, either due to the effects of gender stereotypes about mathematics; or because it is seen as more acceptable for females than males to express anxiety in general. The view that females reporting greater mathematics anxiety might be mainly due to social desirability bias is called into question by the fact that females are also less likely than males to take post‐compulsory mathematics courses and to choose STEM‐related careers. Nevertheless, it would be desirable to have more studies where males and females are compared with regard to objective. physiological measures of anxiety, such as heart rate and skin conductance, in response to numerical stimuli and mathematical tasks.

## GENDER DIFFERENCES: ARE THERE GENDER DIFFERENCES IN THE RELATIONSHIP BETWEEN MATHEMATICS ANXIETY AND PERFORMANCE?

There may be gender differences in the relationship between attitudes and performance. There are, however, some conflicting results as to exactly how these might differ. Devine et al. (2012) gave 433 British 11‐to‐15‐year‐olds mental mathematics tests and also questionnaires measuring mathematics anxiety and test anxiety. Girls and boys did not differ in mathematics performance, but girls showed higher mathematics anxiety and higher test anxiety than boys. Both boys and girls showed a negative correlation between mathematics anxiety and mathematics performance. However, once the researchers controlled for test anxiety, this relationship disappeared for boys, in contrast with some previous findings that suggested a specific relationship between mathematics anxiety and mathematics performance in both genders. Only girls continued to show an independent relationship between mathematics anxiety and mathematics performance after controlling for test anxiety.

Some other studies have also shown a greater link between mathematics anxiety and performance in girls than in boys, usually even without controlling for test anxiety (Erturan & Jansen, 2015; Geary et al., 2019, 2023; Van Mier et al., 2018; Yu et al., 2023). Koponen et al. (2024) found that response to an arithmetic fluency intervention correlated more (negatively) with mathematics anxiety in girls than in boys and more (positively) with domain‐general cognitive skills in boys than in girls. However, some studies have found no gender differences in the relationship between mathematics anxiety and mathematics achievement, and meta‐analyses by Barroso et al. (2021) and Zhang et al. (2019) did not find an overall significant effect. Hembree (1990) and Ma and Xu (2004) obtained results suggesting that mathematics anxiety is more negatively related to achievement in males than in females. Most of the above studies did not, however, control for test anxiety. Also, most studies have not looked at whether there could be gender differences in the relationships between mathematics anxiety and performance on different aspects of mathematics. Miller and Bichsel (2004) did look at this issue in adults and found that mathematics anxiety was more related to basic mathematics scores in males but to applied mathematics scores in females.

It is important to consider whether the general relationship between mathematics anxiety and performance is influenced by gender dynamics. As most studies show a reciprocal bidirectional relationship between mathematics anxiety and mathematics performance, it is possible that gender differences in the relationship between mathematics performance and mathematics anxiety could reflect differences in the extent to which experiences of failure and success affect anxiety, as well as differences in the extent to which anxiety affects performance. For example, might girls, given their greater susceptibility to test anxiety, be more susceptible to the negative effects of failure? Studies in the area have been somewhat conflicting and inconclusive. There are several studies that have found no gender differences in the relationship between mathematics anxiety and performance (Ma, 1999; Meece et al., 1990; Wu et al., 2012). Wang et al. (2020) found gender differences in the relationship between mathematics anxiety and mathematics self‐rating in the opposite direction from what might be predicted: secondary school boys, but not girls, showed a bidirectional longitudinal relationship between mathematics anxiety and self‐rating.

Parental mathematics anxiety may influence their children's mathematics anxiety and/or their performance, and this may be particularly the case for girls. Some studies have suggested that parental mathematics anxiety negatively predicts children's performance (Becker et al., 2022; Simmons et al., 2024), though this may only be the case if parents are highly involved in their children's homework (Maloney et al., 2015) and especially if they experience and express negative emotions while doing so (DiStefano et al., 2020). Other studies have suggested that parental mathematics anxiety predicts their children's mathematics anxiety, but not their actual performance (Casad et al.'s, 2015; Szczygiel, 2020a; Vanbinst et al., 2020), though Simmons et al. (2024) found the reverse. Clearly, much more research is needed in the area.

Gender stereotypes may be communicated to children (Bleeker & Jacobs, 2004; Jacobs, 1991; Tomasetto et al., 2015). Jacobs (1991) found that if fathers had gender stereotypes about mathematics, their sons tended to perform better and their daughters worse at mathematics, as though this created a self‐fulfilling prophecy. If mothers had gender stereotypes about mathematics, both their sons and daughters tended to perform worse at mathematics, perhaps because such mothers are more likely to experience and express mathematics anxiety and negative attitudes toward mathematics.

Thus, most studies suggest that females experience greater mathematics anxiety than males, and that this can be only partially explained by females tending to have greater general anxiety and greater test anxiety than males. It also cannot be explained by actual gender differences in performance, as nowadays there are no such gender differences in most countries. It is likely that exposure to gender stereotypes contributes to the gender differences in mathematics anxiety, though there may also be other, possibly as yet undiscovered, factors involved.

## CULTURAL AND NATIONAL DIFFERENCES IN MATHEMATICS ANXIETY

There is a significant amount of research on national and cultural differences in mathematics anxiety, though there is often a lack of precision about how ‘culture’ should be defined. In practice, most relevant studies involve international comparisons. It must be borne in mind when considering such comparisons that (a) nations are not cultural monoliths, and there may be many cultural differences within a country and (b) the cultural differences that may result in international differences are multifactorial, including socioeconomic factors, cultural attitudes to mathematics, often mediated by parents, teachers and/or peers; educational practices; and actual levels of mathematical achievement.

Levine and Pantoja (2021) have suggested that ‘key socialisers—parents and teachers—hold: general (math‐gender stereotypes and mindsets), self‐relevant (math anxiety) and child‐specific (expectations and value of math for their child or student)’ and cite evidence that these do affect children's anxiety, attitudes and performance. Although most studies of the subject have dealt with the effects of parents’ and teachers’ attitudes within a single culture, it is likely that parents’ and teachers’ attitudes may be a key factor in cultural differences and may contribute to the intergenerational transmission and thus persistence of these cultural differences.

There are two main international surveys which investigate and compare mathematics achievement in different countries. One of these is PISA (Programme for International Student Assessment) and the other is TIMSS (Trends in International Mathematics and Science Study). These surveys are both carried out at intervals. While achievement is always assessed, mathematics anxiety has been assessed in some years but not others. Where it has been studied, mathematics anxiety is generally found to differ significantly between different countries.

Fan et al. (2019) looked at mathematics anxiety in Finnish, Korean and American 15‐year‐old pupils, as assessed in the PISA international comparison of 2012. The American pupils reported the highest levels of mathematics anxiety, and the Finnish pupils the lowest. As regards performance, the Korean pupils did best of the three groups and the American pupils least well.

One study that involved a particularly fine‐grained analysis of international differences in mathematics anxiety, and the factors that may influence it, was carried out by Yuan et al. (2023). They investigated mathematics anxiety as measured by the Student Questionnaire in the 2012 PISA tests in five Asian‐Pacific countries: China (Shanghai), Singapore, South Korea, Malaysia and Indonesia. The first three countries were among the highest scoring in the international comparisons of actual performance, while the two latter scored below the international median. The Shanghai pupils showed the lowest mathematics anxiety, followed by those of Singapore and South Korea, and those of Indonesia and Malaysia showed the highest. In all countries, mathematics anxiety was negatively associated with actual performance and with the level of interest in mathematics. Girls showed higher mathematics anxiety than boys, with the gender differences being large in Shanghai, Singapore and South Korea and small in Malaysia and Indonesia. Socio‐economic status was negatively associated with mathematics anxiety, with the association being strong in Shanghai and Singapore and weaker in the other places. In Shanghai, Singapore and South Korea, mathematics anxiety was negatively associated with a higher evaluation of one's own ability to solve problems, high ratings of the importance of mathematics, and self‐attribution for mathematics performance, but these associations were not found in Malaysian or Indonesian pupils. Parental attitudes and friends' performance were associated with increased mathematics anxiety in Malaysia, Indonesia and South Korea, but had no relation to mathematics anxiety in Shanghai or Singapore.

Lau et al. (2022) investigated data from the TIMSS 2015 Grade 4 and Grade 8 surveys (Martin et al., 2016) and the PISA 2012 survey (OECD, 2014). They found that girls reported more mathematics anxiety than boys and that mathematics anxiety was negatively correlated with pupil ratings of their mathematics teachers' competence and with the amount of homework given. It was also negatively correlated with the teachers' own confidence in teaching mathematics, which may be due to teachers transmitting their own mathematics anxiety to their students, or to their confidence being an accurate reflection of their competence in teaching the subject, and competent teaching leading to reduced mathematics anxiety.

Wei et al. (2024) looked at mathematics performance, mathematics anxiety and executive functions in Italian and Chinese third and fourth graders: a younger group than those usually included in such studies. The Chinese children did better at both exact calculation and arithmetical estimation, and showed lower mathematics anxiety than the Italian children. They also performed better at the executive function of shifting, but the Italian children performed better at visuospatial updating. Notably, although the two groups differed in both mathematics anxiety and arithmetical performance, the negative relationship between the two was very similar in both groups.

Most studies of international differences in mathematics anxiety and their correlates have focussed on relatively high‐income countries, with less attention given to mathematics anxiety in developing countries (Hunt et al., 2021). Those studies that have been carried out in middle‐ and low‐income countries indicate that mathematics anxiety is a significant problem in these countries and is affected by many of the same factors as in better‐off countries. Parental mathematics anxiety is perhaps more consistently found to be related to that of their children. Soni and Kumari (2017) studied almost 600 10‐ to 15‐year‐olds in India and found that their mathematics anxiety and attitudes were correlated with, and probably influenced by, those of their parents. Similar results were obtained in Bangladesh (Haque & Farhana, 2017) and in Turkey (Kesici, 2018; Sarı & Hunt, 2020). These findings can be in part explained by parental mathematics anxiety usually correlating negatively with parental level of education, and parental education level being likely to vary more in developing than developed countries.

## CULTURAL/NATIONAL DIFFERENCES: DO INTERNATIONAL DIFFERENCES IN MATHEMATICS ANXIETY CORRESPOND TO INTERNATIONAL DIFFERENCES IN PERFORMANCE?

International comparisons consistently reveal international differences in mathematics *performance*: in particular, East Asian pupils usually perform better in mathematics than Europaean and American pupils (Mullis, Martin, Foy, & Hooper, 2016; Mullis, Martin, & Loveless, 2016). Is this reflected in mathematics anxiety and other attitudes?

Mathematics anxiety could be related to a country's level of achievement in more ways than one. Children in higher‐achieving countries could have positive attitudes to mathematics because they are doing well (and/or may do well because they have positive attitudes). On the other hand, they could be more anxious about mathematics and rate themselves lower because high‐achieving countries often attach high importance to mathematics and to academic achievement in general, and this may increase academic pressures on children and lead to greater fear of failure. Moreover, such children might rate themselves lower in mathematics because they are comparing themselves with their own peers, who are likely to be high‐achieving.

In fact, studies comparing mathematics anxiety in different countries (Askew et al., 2010; Foley et al., 2017; Lee, 2009) have found inconsistent relationships between a country's overall mathematics achievement level and the average level of mathematics anxiety among children in that country. Overall, there does seem to be some negative relationship between countries' position in international league tables of mathematical performance and their position in tables of mathematics anxiety (OECD, 2023). However, children in high‐achieving East Asian countries, such as Korea and Japan, tend to demonstrate high mathematics anxiety, while those in high‐achieving Western Europaean countries, such as Finland, the Netherlands, Liechtenstein and Switzerland, tend to demonstrate low mathematics anxiety. It is, of course, difficult to tell to what extent responses might be influenced by cultural differences in the perceived social desirability of expressing confidence versus anxiety with regard to academic subjects, and, as in the case of gender, it would be desirable to have more studies using physiological measures of anxiety rather than just self‐report.

## CULTURAL/NATIONAL DIFFERENCES: ARE THERE INTERNATIONAL DIFFERENCES IN THE RELATIONSHIP BETWEEN MATHEMATICS ANXIETY AND PERFORMANCE?

Despite the inconsistency in the relationships between national mathematics achievement and national mathematics anxiety levels, a negative relationship between anxiety and performance has consistently been found *within* any given country (Foley et al., 2017; Lee, 2009; Ma & Kishor, 1997). There is no evidence that the national level of mathematics achievement, or indeed any other national or cultural factor, has a significant influence on the relationship between anxiety and performance.

Lau et al. (2022) found that there was a negative relationship between mathematics anxiety and achievement in almost all countries (Albania was the one country where no such relationship was found). In about half of the countries, the average level of mathematics anxiety reported by pupils within the same school or classroom was related to each individual pupil's mathematics achievement, even after controlling for the individual's level of mathematics anxiety. It is not known why this is the case, or why it was found in some but not all countries. It may reflect peer group influences on the individual pupils, or it may be due to some aspects of the teaching environment influencing anxiety and achievement at both the individual and group levels in some countries.

It would be of interest to have more comparative studies of the relationships between mathematics anxiety, performance and other factors in different countries, in relation to the countries' positions in international comparisons of mathematics achievement. Some studies in China suggest that some of the relationships may not differ greatly from those in other, mostly lower‐scoring countries.

Ching (2017) found that mathematics anxiety in second grade was a significant longitudinal predictor of Chinese children's mathematical performance in third grade, even after controlling for nonverbal IQ, number skills, general anxiety and test anxiety. This was especially true of mathematical tasks that required a lot of cognitive processing and, as in some studies of English‐speaking Western participants, for children with high levels of working memory. This suggests that, as found in some Western studies, mathematics anxiety impairs performance at least in part by disrupting working memory. The greater impact on children with better working memory probably reflects the fact that in this age group, children with better working memory skills are more likely to use strategies, such as mental calculation, which emphasise working memory than children with poorer working memory skills and are therefore more likely to be negatively affected by mathematics anxiety.

Some studies have looked at possible interactions between the effects of culture and of gender. Gender differences in self‐reported mathematics anxiety and other negative attitudes are found to occur in many countries (Sarfo et al., 2020), and do not seem to be related to actual gender differences in performance in the different countries. Ismail and Awang (2008) studied Malaysian eighth‐grade students at a time when Malaysian girls had recently performed significantly better than boys in the TIMSS assessment. They were not asked about mathematics anxiety directly, but they were asked to rate their own ability in mathematics. Boys rated themselves significantly higher than girls did.

Thus, there are definite cultural differences in mathematics anxiety, but we do not as yet have a clear understanding of what causes them. One problem is that most cross‐cultural studies have focussed on differences between different countries, and culture is not identical to nationality. Most studies of cultural differences in mathematics within a country or indeed between countries have not focussed on mathematics anxiety. There is much more research that needs to be done in this area.

## FURTHER QUESTIONS

Despite the large amount of research that has been carried out on mathematics anxiety, much more research still needs to be done. Many important questions are yet to be adequately answered and are appearing particularly important, especially given the increasing evidence that mathematics anxiety cannot be seen in simple terms, but depends on multiple factors (e.g. see Szucs and Mammarella's (2024) biopsychological–social model). For example, it is important to investigate effects of *context* on mathematics anxiety; to investigate the influences of environmental factors on age differences; and to investigate the combined effects of age, gender and culture in the same studies.

### Context

1. To what extent do people exhibit mathematics anxiety outside academic contexts? Cipora et al. (2022) point out that, with a few exceptions (e.g., Hart & Ganley, 2019) few studies have focussed on mathematics anxiety outside academic contexts. Yet this is very important to study, as mathematics anxiety is likely to be detrimental in many practical, occupational and financial situations.
2. More specifically, to what extent does mathematics anxiety differ for similar mathematical activities in different contexts? Marked differences in strategies and performance have been found, for example, in Brazilian child street traders working out similar arithmetic problems in a school context and a street market context (Carraher et al., 1985) and in Californian housewives working out similar proportional reasoning problems in a formal context and when choosing good bargains when shopping (Lave, 1988). Banerjee et al. (2025) studied child market traders in India and obtained similar results to Carraher et al. (1985), finding that the children performed much better on market problems than on arithmetically similar problems presented in the more abstract form typical of school mathematics. The reverse was the case for schoolchildren who did not work in markets: they performed much better on school‐type mathematics problems than on market problems. Would such differences between contexts be reflected in levels of mathematics anxiety? Would people show less anxiety in informal than formal contexts, because the former are less associated with school performance evaluations and examinations? Or would people be less anxious in contexts that are familiar to them, whether these are formal or informal?

### Age

3 Moving from context to age, given that mathematical ability consists of multiple components (Dowker, 2015, 2023), to what extent does mathematics anxiety differ between different components? In this connection, to what extent is the often‐observed increase in mathematics anxiety with age during the school years caused by changes in the contents of the mathematics curriculum as pupils get older? One ambiguity that arises in most studies of age differences is that age is inextricably linked to stage of schooling, so that apparent age increases in mathematics anxiety could really be the result of increased anxiety as the content of the mathematics curriculum becomes more abstract or more demanding of working memory.
4 To what extent are age differences, and perhaps also gender, and cultural differences in mathematics anxiety, due to effects of (a) parental anxiety; (b) teachers' anxiety; (c) attitudes and anxiety in the wider culture; (d) experience of tests and examinations?

### Combining age, gender and culture

5 To what extent, and in what ways, do the effects of age, gender and culture interact in influencing mathematics anxiety and in explaining its causes and its relationships to performance? There are few studies that combine investigations of all three, and such studies would be an important step to further understanding/.

## CONCLUSIONS

Mathematics anxiety is a significant problem for many individuals. There is somewhat conflicting evidence regarding exactly how frequent it is, but even the lower estimates suggest that it affects a very large number of people. There is a bidirectional relationship between mathematics anxiety and performance, which highlights the importance of the issue. Most studies suggest that mathematics anxiety increases with age during childhood, and most, though not all, suggest that the relationship between mathematics anxiety and performance increases with age. Most studies also indicate that females experience greater mathematics anxiety than males, which seems at least in part to be due to exposure to gender stereotypes in society, though other factors may also contribute. There also seem to be cultural and national differences in levels of mathematics anxiety, which do not completely correspond to national differences in mathematics performance. Much more research is needed if we are to fully understand and explain what factors influence mathematics anxiety, and thus how it might be prevented or ameliorated.

## IMPLICATIONS FOR PRACTICE

Most studies indicate that mathematics anxiety is relatively common; that it often makes mathematics learning a stressful experience; and that it correlates negatively with performance, both in order to improve people's mathematics achievement, and in order to reduce their negative emotional experiences, it is desirable to reduce mathematics anxiety as much as possible.

There are a number of interventions that have been found to be helpful for students with mathematics anxiety. Supekar et al. (2015) found that am intensive individualised 8‐week, tutoring programme in addition and subtraction reduced mathematics anxiety, in a sample of 7‐to 9‐year‐old children. They explained their findings mainly in terms of desensitisation. An alternative explanation could be that the intervention improved mathematical performance, and thus worked on the performance‐to‐anxiety pathway of the reciprocal relationship between anxiety and performance.

It also appears that writing out one's worries before doing a task or test may reduce state anxiety and certainly reduces the negative impact of anxiety on performance. This was found by Ramirez and Beilock (2011) for test anxiety in general, and by Park et al. (2014) specifically for mathematics anxiety. It is likely that expressive writing disrupts the negative emotional cognitions and ruminations, and this reduces their negative impact on the working memory resources needed for problem solving. This method is not, of course, suitable for very young children with limited writing skills, but appears promising for older children and adults.

There are also some newer treatments that are being tried: for example, application of transcranial direct current stimulation (tDCS) to the dorsolateral prefrontal cortex (Sarkar et al., 2014). In Sarkar et al.'s (2014) study, participants were given tDCS via a battery‐driven stimulator using rubber electrodes. The stimulation appeared to have a positive effect, compared with sham stimulation, on people with high mathematics anxiety, who showed faster reaction times in simple arithmetic decisions and reduced concentrations of the stress hormone cortisol. However, it had the reverse effect on people who started with low mathematics anxiety, who showed slower reaction times in simple arithmetic decisions and less decrease in cortisol concentration than those who received sham stimulation. There needs to be more research in the area before we can draw firm conclusions about the effectiveness of this form of treatment.

For comprehensive discussion of currently available interventions, see Codding et al. (2023); Kirkland and Hunt (2025) and Sidney et al. (2024), Here, we will focus specifically on the implications for practice of the findings about the effects of the factors of age, gender and culture?

1. With regard to *age*, we know that mathematics anxiety can be found in young children but that it increases with age, This means both that it is desirable to watch for signs of mathematics anxiety even in the early years, and that it is desirable to intervene to prevent or ameliorate mathematics anxiety as early as possible, before it intensifies with age, and before the reciprocal anxiety‐performance links have led to a vicious circle of increasing anxiety and worsening relative perf
2. With regard to *gender*, studies suggest that stereotyping – both explicit (“Maths is hard for girls”) and implicit (e.g. lack of reference in children's books and TV programmes to girls and women doing mathematics) ‐ should be avoided. Building up mathematical confidence in mothers and female teachers may help to reduce intergenerational transmission of mathematic anxiety, Moreover, measures that reduce test anxiety in general‐ reducing the dependence of secondary and university qualifications on high‐stakes timed tests‐ are likely to be particularly beneficial to girls, who are subject to both test anxiety and mathematics anxiety.
3. With regard to culture/ nationality, we are hampered in our knowledge both by the fact that international mathematics anxiety studies often do not distinguish clearly between the influences of culture, nation and region, and by the fact that there has been less research regarding some countries (especially developing countries) than others. However, clear conclusions include the fact that mathematics anxiety is negatively related to performance almost everywhere: i.e. this reciprocal relationship is not culture‐dependent. This provides further evidence for the importance of early interventions with both mathematics anxiety and mathematical difficulties, to reduce the chance of a vicious circle developing. It is important to find ways of developing relatively non‐intensive interventions, which are feasible in low‐and middle‐income countries and in under‐resourced areas in higher‐income countries. Reducing mathematics anxiety may not only be desirable for individuals, but may lead to desirable cultural change by reducing mathematics anxiety in future generations, given that intergenerational transmission of mathematics anxiety is a significant factor (Hunt et al., 2021), perhaps especially in lower‐income countries.

## AUTHOR CONTRIBUTIONS

**Ann Dowker:** Writing – review and editing; writing – original draft; conceptualization; methodology; investigation.

## CONFLICT OF INTEREST STATEMENT

The author declares no conflict of interest.

## Data Availability

The data that support the findings of this study are available on request from the corresponding author. The data are not publicly available due to privacy or ethical restrictions.

## References

[bjep70057-bib-0001] Abín, A. , Núñez, J. C. , Rodríguez, C. , Cueli, M. , García, T. , & Rosário, P. (2020). Predicting mathematics achievement in secondary education: The role of cognitive, motivational, and emotional variables. Frontiers in Psychology, 11, 876. 10.3389/fpsyg.2020.00876B 32528351 PMC7264990

[bjep70057-bib-0002] Aldrup, K. , Klusmann, U. , & Lüdtke, O. (2020). Reciprocal associations between students' mathematics anxiety and achievement: Can teacher sensitivity make a difference? Journal of Educational Psychology, 112(4), 735–750. 10.1037/edu0000398

[bjep70057-bib-0003] Alken, L. R. (1974). Two scales of attitude toward mathematics. Journal for Research in Mathematics Education, 5(2), 67–71. 10.2307/748616

[bjep70057-bib-0004] Ambady, N. , Shih, M. , Kim, A. , & Pittinsky, T. L. (2001). Stereotype susceptibility in children: Effects of identity activation on quantitative performance. Psychological Science, 12, 385–390. 10.1111/1467-9280.00371 11554671

[bjep70057-bib-0005] Ashcraft, M. H. (2002). Math anxiety: Personal, educational, and cognitive consequences. Current Directions in Psychological Science, 11(5), 181–185. 10.1111/1467-8721.00196

[bjep70057-bib-0006] Ashcraft, M. H. , Kirk, E. P. , & Hopko, D. (1998). On the cognitive consequences of mathematics anxiety. In C. Donlan (Ed.), The development of mathematical skills (pp. 175–196). Erlbaum.

[bjep70057-bib-0007] Ashcraft, M. H. , & Moore, A. M. (2009). Mathematics anxiety and the affective drop in performance. Journal of Psychoeducational Assessment, 27, 197–205. 10.1177/0734282908330580

[bjep70057-bib-0008] Ashcraft, M. H. , & Ridley, K. S. (2005). Math anxiety and its cognitive consequences: A tutorial review. In Handbook of mathematical cognition (pp. 315–327). Psychology Press.

[bjep70057-bib-0009] Askew, M. , Hodgen, J. , Hossain, S. , & Bretscher, N. (2010). Values and variables: Mathematics education in high‐performing countries. Nuffield Foundation.

[bjep70057-bib-0010] Avancini, C. , & Szucs, D. (2019). Psychophysiological correlates of mathematics anxiety. In I. C. Mammarella , S. Caviola , & A. Dowker (Eds.), Mathematics anxiety: What is known and what is still to be understood (pp. 42–61). Routledge. 10.4324/9780429199981

[bjep70057-bib-0011] Banerjee, A. V. , Bhattacharjee, S. , Chattopadhyay, R. , Duflo, E. , Ganimian, A. J. , Rajah, K. , & Spelke, E. S. (2025). Children's arithmetic skills do not transfer between applied and academic mathematics. Nature, 639(8055), 673–681. 10.1038/s41586-024-08502-w 39910295 PMC11922737

[bjep70057-bib-0012] Barroso, C. , Ganley, C. M. , McGraw, A. L. , Geer, E. A. , Hart, S. A. , & Daucourt, M. C. (2021). A meta‐analysis of the relation between math anxiety and math achievement. Psychological Bulletin, 147, 134–168. 10.1037/bul0000307 33119346 PMC8300863

[bjep70057-bib-0013] Batchelor, S. , Torbeyns, J. , & Verschaffel, L. (2019). Affect and mathematics in young children: An introduction. Educational Studies in Mathematics, 100(3), 201–209. 10.1007/s10649-018-9864-x

[bjep70057-bib-0014] Becker, M. , Litkowski, E. , Duncan, R. J. , & Schmitt, S. A. (2022). Parents' math anxiety and mathematics performance of pre‐kindergarten children. Journal of Experimental Child Psychology, 214, 105302. 10.1016/j.jecp.2021.105302 34624707

[bjep70057-bib-0015] Beilock, S. L. , Gunderson, E. A. , Ramirez, G. , & Levine, S. C. (2010). Female teachers' math anxiety affects girls' math achievement. Proceedings of the National Academy of Sciences, 107, 1860–1863. 10.1073/pnas.0910967107 PMC2836676 20133834

[bjep70057-bib-0016] Beilock, S. L. , Rydell, R. J. , & McConnell, A. R. (2007). Stereotype threat and working memory: Mechanisms, alleviation, and spillover. Journal of Experimental Psychology: General, 136(2), 256–276. 10.1037/0096-3445.136.2.256 17500650

[bjep70057-bib-0017] Bleeker, M. , & Jacobs, J. E. (2004). Achievement in math and science: Do mothers' beliefs matter 12 years later? Journal of Educational Psychology, 96, 97–109. 10.1037/0022-0663.96.1.97

[bjep70057-bib-0018] Breckler, S. J. (1984). Empirical validation of affect, behavior, and cognition as distinct components of attitude. Journal of Personality and Social Psychology, 47(6), 1191–1205. 10.1037//0022-3514.47.6.1191 6527214

[bjep70057-bib-0019] Brown, M. , Brown, P. , & Bibby, T. (2010). “I would rather die”: Reasons given by 16 year‐olds for not continuing their study in mathematics. Research in Mathematics Education, 10, 3–18. 10.1080/14794800801915814

[bjep70057-bib-0020] Cain‐Caston, M. (1993). Parent and student attitudes toward mathematics as they relate to third grade mathematics achievement. Journal of Instructional Psychology, 20(2), 96–101.

[bjep70057-bib-0021] Callan, S. (2015). The fear factor . http://mathsaction.org.uk/

[bjep70057-bib-0022] Carey, E. , Devine, A. , Hill, F. , Dowker, A. , McLellan, R. , & Szucs, D. (2019). Understanding mathematics anxiety: Investigating the experiences of UK primary and secondary school students. Centre for Neuroscience in Education. 10.17863/CAM.37744

[bjep70057-bib-0024] Carey, E. , Hill, F. , Devine, A. , & Szucs, D. (2016). The chicken or the egg? The direction of the relationship between mathematics anxiety and mathematics performance. Frontiers in Psychology, 6, 1987. 10.3389/fpsyg.2015.01987 26779093 PMC4703847

[bjep70057-bib-0025] Cargnelutti, E. , Tomasetto, C. , & Passolunghi, M. C. (2017). How is anxiety related to math performance in young students? A longitudinal study of grade 2 to grade 3 children. Cognition and Emotion, 31(4), 755–764. 10.1080/02699931.2016.1147421 26935005

[bjep70057-bib-0026] Casad, B. J. , Hale, P. , & Wachs, F. L. (2015). Parent‐child math anxiety and math‐gender stereotypes predict adolescents' math education outcomes. Frontiers in Psychology, 6, 1597. 10.3389/fpsyg.2015.01597 26579000 PMC4630312

[bjep70057-bib-0027] Caviola, S. , Toffalini, E. , Giofre, D. , Ruiz, J. M. , Szucs, D. , & Mammarella, I. C. (2022). Math performance and academic anxiety forms, from sociodemographic to cognitive aspects: A meta‐analysis on 906,311 participants. Educational Psychology Review, 34(1), 363–399. 10.1007/s10648-021-09618-5

[bjep70057-bib-0028] Chapman, B. P. , Duberstein, P. R. , Sörensen, S. , & Lyness, J. M. (2007). Gender differences in five factor model personality traits in an elderly cohort: Extension of robust and surprising findings to an older generation. Personality and Individual Differences, 43, 1594–1603. 10.1016/j.paid.2007.04.028 18836509 PMC2031866

[bjep70057-bib-0029] Ching, B. H.‐H. (2017). Mathematics anxiety and working memory: Longitudinal associations with mathematical performance in Chinese children. Contemporary Educational Psychology, 51, 99–113. 10.1016/j.cedpsych.2017.06.006

[bjep70057-bib-0030] Chinn, S. (2009). Mathematics anxiety in secondary students in England. Dyslexia, 15, 61–68. 10.1002/dys.381 19089884

[bjep70057-bib-0031] Chipman, S. F. , Krantz, D. H. , & Silver, R. (1992). Mathematics anxiety and science careers among able college women. Psychological Science, 3, 292–295. 10.1111/j.1467-9280.1992.tb00675.x

[bjep70057-bib-0032] Cipora, K. , Artemenko, C. , & Nuerk, H.‐C. (2019). Different ways to measure math anxiety. In I. Mammarella , S. Caviola , & A. Dowker (Eds.), Mathematics anxiety: What is known and what is still to be understood (pp. 20–41). Routledge. 10.4324/9780429199981

[bjep70057-bib-0033] Cipora, K. , Santos, F. H. , Kucian, K. , & Dowker, A. (2022). Mathematics anxiety — Where are we and where shall we go? Annals of the New York Academy of Sciences, 1513(1), 10–20. 10.1111/nyas.14770 35322431 PMC9542812

[bjep70057-bib-0034] Codding, R. S. , Goodridge, A. E. , Hill, E. , Kromminga, K. R. , Chehayeb, R. , Volpe, R. J. , & Scheman, N. (2023). Meta‐analysis of skill‐based and therapeutic interventions to address math anxiety. Journal of School Psychology, 100, 101229. 10.1016/j.jsp.2023.101229 37689437

[bjep70057-bib-0035] Costa, P. T. , Terracciano, A. , & McCrae, R. R. (2001). Gender differences in personality traits across cultures: Robust and surprising findings. Journal of Personality and Social Psychology, 81, 322–331. 10.1037/0022-3514.81.2.322 11519935

[bjep70057-bib-0036] Devine, A. , Fawcett, K. , Szucs, D. , & Dowker, A. (2012). Gender differences in mathematics anxiety and the relation to mathematics performance while controlling for test anxiety. Behavioral and Brain Functions, 8, 33. 10.1186/1744-9081-8-33 22769743 PMC3414752

[bjep70057-bib-0037] Devine, A. , Hill, F. , Carey, E. , & Szűcs, D. (2018). Cognitive and emotional math problems largely dissociate: Prevalence of developmental dyscalculia and mathematics anxiety. Journal of Educational Psychology, 110(3), 431–444. 10.1037/edu0000222

[bjep70057-bib-0038] Dew, K. H. , Galassi, J. P. , & Galassi, M. D. (1984). Math anxiety: Relation with situational test anxiety, performance, physiological arousal, and math avoidance behavior. Journal of Counseling Psychology, 31, 580–583. 10.1037/0022-0167.31.4.580

[bjep70057-bib-0039] DiStefano, M. , O'Brien, B. , Storozuk, A. , Ramirez, G. , & Maloney, E. A. (2020). Exploring math anxious parents' emotional experience surrounding math homework‐help. International Journal of Educational Research, 99, 101526. 10.1177/0956797615592630

[bjep70057-bib-0040] Dossey, A. , Mullis, I. V. S. , Lindquist, M. M. , & Chambers, D. L. (1988). The mathematics report card. Are we measuring up? Trends and achievement based on the 1986 National Assessment. Educational Testing Service.

[bjep70057-bib-0041] Dowker, A. , Bennett, K. , & Smith, L. (2012). Attitudes to mathematics in primary school children. Child Development Research, 2012, 124939. 10.1155/2012/124939

[bjep70057-bib-0042] Dowker, A. , Cheriton, O. , & Horton, R. (2022). Age differences in attitudes to mathematics . Paper presented at the 16th International Conference of The Mathematics Education for the Future Project: Building on the Past to Prepare for the Future, King's College, Cambridge University, UK, August 8–13, 2022.

[bjep70057-bib-0043] Dowker, A. , Cheriton, O. , & Horton, R. (2023). What factors influence mathematics anxiety?: Gender, age and culture . Keynote Speech and 42nd Vernon Wall Lecture, British Psychological Society Education Section Conference, Liverpool, September 14th, 2023.

[bjep70057-bib-0044] Dowker, A. , Cheriton, O. , Horton, R. , & Mark, W. (2019). Relationships between attitudes and performance in young children's mathematics. Educational Studies in Mathematics, 100(3), 211–230. 10.1007/s10649-019-9880-5

[bjep70057-bib-0045] Dowker, A. , Sarkar, A. , & Looi, C. Y. (2016). Mathematics anxiety: What have we learned in 60 years? Frontiers in Psychology, 7, 508. 10.3389/fpsyg.2016.00508 27199789 PMC4842756

[bjep70057-bib-0046] Dreger, R. M. , & Aiken, L. R. (1957). The identification of number anxiety in a college population. Journal of Educational Psychology, 48, 344–351. 10.1037/h0045894

[bjep70057-bib-0047] Du, C. , Qin, K. , Wang, Y. , & Xin, T. (2021). Mathematics interest, anxiety, self‐efficacy and achievement: Examining reciprocal relations. Learning and Individual Differences, 9, 102060. 10.1016/j.lindif.2021.102060

[bjep70057-bib-0048] Else‐Quest, N. , Hyde, J. S. , & Linn, M. (2010). Cross‐national patterns of gender differences in mathematics: A meta‐analysis. Psychological Bulletin, 136, 103–127. 10.1037/a0018053 20063928

[bjep70057-bib-0049] Erturan, S. , & Jansen, B. (2015). An investigation of boys' and girls' emotional experience of math, their math performance, and the relation between these variables. European Journal of Psychology of Education, 30(4), 421–435. 10.1007/s10212-015-0248-7

[bjep70057-bib-0050] Fan, X. , Hambleton, R. K. , & Zhang, M. (2019). Profiles of mathematics anxiety among 15‐year‐old students: A cross‐cultural study using multi‐group latent profile analysis. Frontiers in Psychology, 10, 9. 10.3389/fpsyg.2019.01217 31191408 PMC6546821

[bjep70057-bib-0051] Faust, M. W. , Ashcraft, M. H. , & Fleck, D. E. (1996). Mathematics anxiety effects in simple and complex addition. Mathematical Cognition, 2, 25–62. 10.1080/135467996387534

[bjep70057-bib-0052] Feingold, A. (1994). Gender differences in personality: A meta‐analysis. Psychological Bulletin, 116, 429–456. 10.1037/0033-2909.116.3.429 7809307

[bjep70057-bib-0053] Fennema, E. , & Sherman, J. A. (1976). Fennema‐Sherman mathematics attitudes scales: Instruments designed to measure attitudes toward the learning of mathematics by females and males. Journal for Research in Mathematics Education, 7, 324–326. 10.2307/748467

[bjep70057-bib-0054] Ferdinand, R. , Malanchini, M. , & Rimfeld, K. (2024). Mathematics interest, self‐efficacy, and anxiety predict STEM career choice in emerging adulthood. Npj Science of Learning, 9, 12. 10.1038/s41539-024-00275-1 39537658 PMC11561120

[bjep70057-bib-0055] Flessati, S. L. , & Jamieson, J. (1991). Gender differences in mathematics anxiety: An artifact of response bias? Anxiety Research, 3(4), 303–312. 10.1080/08917779108248759

[bjep70057-bib-0056] Flore, P. C. , & Wicherts, J. M. (2015). Does stereotype threat influence performance of girls in stereotyped domains? A meta‐analysis. Journal of School Psychology, 53(1), 25–44. 10.1016/j.jsp.2014.10.002 25636259

[bjep70057-bib-0057] Foley, A. E. , Herts, J. B. , Borgonovi, F. , Guerriero, S. , Levine, S. C. , & Beilock, S. L. (2017). The math anxiety‐performance link: A global phenomenon. Current Directions in Psychological Science, 26(1), 52–58. 10.1177/0963721416672463

[bjep70057-bib-0058] Galdi, S. , Cadinu, M. , & Tomasetto, C. (2014). The roots of stereotype threat: When automatic associations disrupt girls' math performance. Child Development, 85(1), 250–263. 10.1111/cdev.12128 23713580

[bjep70057-bib-0059] Gattas, S. (2024). Multidimensional mechanisms underlying numeracy development: The role of affect in the relationship between executive functions and foundational stages of numeracy development. D. Phil thesis, University of Oxford.

[bjep70057-bib-0060] Geary, D. C. , Hoard, M. K. , Nugent, L. , Chu, F. W. , Scofield, J. E. , & Hibbard, D. F. (2019). Sex differences in mathematics anxiety and attitudes: Concurrent and longitudinal relations to mathematical competence. Journal of Educational Psychology, 111(8), 1447–1461. 10.1037/edu0000355 31768075 PMC6876627

[bjep70057-bib-0061] Geary, D. C. , Hoard, M. K. , Nugent, L. , Ünal, Z. E. , & Green, N. R. (2023). Sex differences and similarities in relations between mathematics achievement, attitudes, and anxiety: A seventh‐to‐ninth grade longitudinal study. Journal of Educational Psychology, 115, 767–782. 10.1037/edu0000793 37928445 PMC10624155

[bjep70057-bib-0062] Gunderson, E. A. , Park, D. , Maloney, E. A. , Beilock, S. L. , & Levine, S. C. (2018). Reciprocal relations among motivational frameworks, math anxiety, and math achievement in early elementary school. Journal of Cognition and Development, 19(1), 21–46. 10.1080/15248372.2017.1421538

[bjep70057-bib-0063] Haque, M. , & Farhana, K. (2017). Relationship between parents' attitude towards math and children's math anxiety. Journal of Child and Adolescent Behavior, 5(4). 10.4172/2375-4494.1000354

[bjep70057-bib-0064] Harari, R. R. , Vukovic, R. K. , & Bailey, S. P. (2013). Mathematics anxiety in young children: An exploratory study. Journal of Experimental Education, 81, 538–555. 10.1080/00220973.2012.727888

[bjep70057-bib-0065] Hart, S. A. , & Ganley, C. M. (2019). The nature of math anxiety in adults: Prevalence and correlates. Journal of Numerical Cognition, 5, 122–139. 10.5964/jnc.v5i2.195 33842689 PMC8034611

[bjep70057-bib-0066] Hembree, R. (1990). The nature, effects, and relief of mathematics anxiety. Journal for Research in Mathematics Education, 21, 33–46. 10.2307/74945

[bjep70057-bib-0068] Hopko, D. R. , Hunt, M. K. , & Armento, M. E. A. (2005). Attentional task aptitude and performance anxiety. International Journal of Stress Management, 12(4), 389–408. 10.1037/1072-5245.12.4.389

[bjep70057-bib-0069] Huguet, P. , & Régner, I. (2009). Counter‐stereotypic beliefs in math do not protect school girls from stereotype threat. Journal of Experimental Social Psychology, 45(4), 1024–1027. 10.1016/j.jesp.2009.04.029

[bjep70057-bib-0070] Hunt, T. E. , Bhardwa, J. , & Sheffield, D. (2017). Mental arithmetic performance, physiological reactivity and mathematics anxiety amongst U.K. primary school children. Learning and Individual Differences, 57, 129–132. 10.1016/j.lindif.2017.03.016

[bjep70057-bib-0071] Hunt, T. E. , Simms, V. , Cahoon, A. , & Muwonge, C. M. (2021). Socio‐cognitive‐affective barriers to mathematics education in developing nations. In W. Leal Filho , P. Gökçin Özuyar , A. Lange Salvia , A. Marisa Azul , L. Londero Brandli , & T. Wall (Eds.), Encyclopaedia of the UN sustainable development goals: Quality education. Springer. 10.1007/978-3-319-69902-8_128-1

[bjep70057-bib-0072] Hyde, J. S. , Lindberg, S. M. , Linn, M. C. , Ellis, A. B. , & Williams, C. C. (2008). Gender similarities characterize math performance. Science, 321(5888), 494–495. 10.1126/science:1160364 18653867

[bjep70057-bib-0073] Ismail, N. A. , & Awang, H. (2008). Differentials in mathematics achievement among eighth‐grade students in Malaysia. International Journal of Science and Mathematics Education, 6, 559–571. 10.1007/s10763-007-9109-4

[bjep70057-bib-0074] Jacobs, J. E. (1991). Influences of gender stereotypes on parent and child mathematics attitudes. Journal of Educational Psychology, 83, 518–527. 10.1037/0022-0663.83.4.518

[bjep70057-bib-0075] Jameson, M. M. (2014). Contextual factors related to math anxiety in second‐grade children. Journal of Experimental Education, 82, 518–536. 10.1080/00220973.2013.813367

[bjep70057-bib-0076] Johnson, D. R. , & Gronlund, S. D. (2009). Individuals lower in working memory capacity are particularly vulnerable to anxiety's disruptive effect on performance. Anxiety, Stress and Coping, 22, 201–213. 10.1080/10615800802291277 18785033

[bjep70057-bib-0077] Johnston‐Wilder, S. , Brindley, J. , & Dent, P. (2014). A survey of mathematics anxiety and mathematical resilience among existing apprentices. Gatsby Charitable Foundation.

[bjep70057-bib-0078] Kersey, A. J. , Braham, E. J. , Csumitta, K. D. , Libertus, M. E. , & Cantlon, J. F. (2018). No intrinsic gender differences in children's earliest numerical abilities. Npj Science of Learning, 3(1), 12. 10.1038/s41539-018-0028-7 30631473 PMC6220191

[bjep70057-bib-0079] Kersey, A. J. , Csumitta, K. D. , & Cantlon, J. F. (2019). Gender similarities in the brain during mathematics development. Npj Science of Learning, 4(1), 19. 10.1038/s41539-019-0057-x 31728205 PMC6841948

[bjep70057-bib-0080] Kesici, A. (2018). Matematik kaygisi ebeveynlerden cocuklara aktarilan kulturel bir miras mi? [is math anxiety a cultural heritage transferred from parents to children?] Dicle Üniversitesi Sosyal Bilimler Enstitüsü Dergisi, 10(20), 304–313.

[bjep70057-bib-0081] Kirkland, H. , & Hunt, T. (2025). Maths anxiety: Solving the equation. Routledge.

[bjep70057-bib-0082] Koponen, T. , Aro, T. , Leskinen, M. , Peura, P. , Viholainen, H. , & Aro, M. (2024). Cognitive skills, math‐related emotions, and beliefs explaining response to arithmetic fluency intervention. The Journal of Experimental Education, 92(3), 411–430. 10.1080/00220973.2023.221921

[bjep70057-bib-0083] Krinzinger, H. , Kaufmann, L. , & Willmes, K. (2009). Math anxiety and math ability in early primary school years. Journal of Psychoeducational Assessment, 27, 206–225. 10.1177/0734282908330583 20401159 PMC2853710

[bjep70057-bib-0084] Kyttala, M. , & Bjorn, P. M. (2010). Prior mathematics achievement, cognitive appraisals and anxiety as predictors of Finnish students' later mathematics performance and career orientation. Educational Psychology, 30(4), 431–448. 10.1080/01443411003724491

[bjep70057-bib-0085] Lau, N. T. T. , Hawes, Z. , Tremblay, P. , & Ansari, D. (2022). Disentangling the individual and contextual effects of math anxiety: A global perspective. Proceedings of the National Academy of Sciences of the United States of America, 119(7), e2115855119. 10.1073/pnas.2115855119 35131942 PMC8851526

[bjep70057-bib-0086] Lave, J. (1988). Cognition in action. Cambridge University Press.

[bjep70057-bib-0087] Lee, J. (2009). Universals and specifics of math self‐concept, math self‐efficacy, and math anxiety across 41 PISA 2003 participating countries. Learning and Individual Differences, 19, 355–365. 10.1016/j.lindif.2008.10.009

[bjep70057-bib-0088] Levine, S. C. , & Pantoja, N. (2021). Development of children's math attitudes: Gender differences, key socializers, and intervention approaches. Developmental Review, 62, 100997. 10.1016/j.dr.2021.100997

[bjep70057-bib-0089] Levy, H. E. , Fares, L. , & Rubinsten, O. (2021). Math anxiety affects females' vocational interests. Journal of Experimental Child Psychology, 210, 105214. 10.1016/j.jecp.2021.105214 34198037

[bjep70057-bib-0090] Lievore, R. , Caviola, S. , & Mammarrella, I. (2024). How trait and state mathematics anxiety could affect performance: Evidence from children with and without specific learning disorders. Learning and Individual Differences, 112, 102459. 10.1016/j.lindif.2024.102459

[bjep70057-bib-0091] Luo, W. , Hogan, D. , Tan, L. S. , Kaur, B. , Ng, P. T. , & Chan, M. (2014). Self‐construal and students' math self‐concept, anxiety and achievement: An examination of achievement goals as mediators. Asian Journal of Social Psychology, 17, 184–195. 10.1111/ajsp.12058

[bjep70057-bib-0092] Ma, X. (1999). A meta‐analysis of the relationship between anxiety toward mathematics and achievement in mathematics. Journal for Research in Mathematics Education, 30, 520–540. 10.2307/749772

[bjep70057-bib-0093] Ma, X. , & Kishor, N. (1997). Assessing the relationship between attitude toward mathematics and achievement in mathematics: A meta‐analysis. Journal for Research in Mathematics Education, 28, 26–47. 10.2307/749662

[bjep70057-bib-0094] Ma, X. , & Xu, J. (2004). Determining the causal ordering between attitude toward mathematics and achievement in mathematics. American Journal of Education, 110, 256–280. 10.1086/383074

[bjep70057-bib-0095] Maloney, E. A. , Ansari, D. , & Fugelsang, J. A. (2011). The effect of mathematics anxiety on the processing of numerical magnitude. Quarterly Journal of Experimental Psychology, 64, 10–16. 10.1080/17470218.2010.533278 21113858

[bjep70057-bib-0096] Maloney, E. A. , & Beilock, S. L. (2012). Math anxiety: Who has it, why it develops, and how to guard against it. Trends in Cognitive Science, 16, 404–406. 10.1016/j.tics.2012.06.008 22784928

[bjep70057-bib-0097] Maloney, E. A. , Ramirez, G. , Gunderson, E. A. , Levine, S. C. , & Beilock, S. L. (2015). Intergenerational effects of parents' math anxiety on children's math achievement and anxiety. Psychological Science, 26, 1480–1488. 10.1177/0956797615592630 26253552

[bjep70057-bib-0098] Mammarella, I. C. , Caviola, S. , Rossi, S. , Patron, E. , & Palomba, D. (2023). Multidimensional components of (state) mathematics anxiety: Behavioral, cognitive, emotional, and psychophysiological consequences. Annals of the New York Academy of Sciences, 1523(1), 91–103. 10.1111/nyas.14982 36964993

[bjep70057-bib-0099] Martin, M. , Mullis, I. V. , & Hooper, I. M. (2016). Methods and procedures in TIMSS 2015. TIMSS & PIRLS International Study Center, Lynch School of Education, Boston College and International Association for the Evaluation of Educational Achievement.

[bjep70057-bib-0100] Mattarella‐Micke, A. , Mateo, J. , Kozak, M. N. , Foster, K. , & Beilock, S. L. (2011). Choke or thrive? The relation between salivary cortisol and math performance depends on individual differences in working memory and math‐anxiety. Emotion, 11(4), 1000–1005. 10.1037/a0023224 21707166

[bjep70057-bib-0101] McLean, C. P. , Asnaani, A. , Litz, B. T. , & Hofmann, S. G. (2011). Gender differences in anxiety disorders: Prevalence, course of illness, comorbidity and burden of illness. Journal of Psychiatric Research, 45, 1027–1035. 10.1016/j.jpsychires.2011.03.006 21439576 PMC3135672

[bjep70057-bib-0102] Meece, J. L. , Wigfield, A. , & Eccles, J. S. (1990). Predictors of math anxiety and its influence on young adolescents' course enrollment intentions and performance in mathematics. Journal of Educational Psychology, 82, 60–70. 10.1037/00220663.82.1.60

[bjep70057-bib-0103] Miller, H. , & Bichsel, J. (2004). Anxiety, working memory, gender, and math performance. Personality and Individual Differences, 37, 591–606. 10.1016/j.paid.2003.09.029

[bjep70057-bib-0105] Mullis, I. V. S. , Martin, M. O. , Foy, P. , & Hooper, M. (2016). TIMSS 2015 international results in mathematics. International TIMSS and PIRLS Study Centre.

[bjep70057-bib-0106] Mullis, I. V. S. , Martin, M. O. , & Loveless, T. (2016). 20 years of TIMSS: International trends in mathematics and science achievement, curriculum, and instruction. International TIMSS and PIRLS Study Centre.

[bjep70057-bib-0107] Namkung, J. M. , Peng, P. , & Lin, X. (2019). The relation between mathematics anxiety and mathematics performance among school‐aged students: A meta‐analysis. Review of Educational Research, 89, 459–496. 10.3102/0034654319843494

[bjep70057-bib-0108] OECD . (2014). PISA 2012 technical report. OECD Publishing.

[bjep70057-bib-0109] OECD . (2023). PISA 2022 Results (Volume I). OECD Publishing.

[bjep70057-bib-0110] Pei, Y. , Poon, K. K. , & Suen, A. (2025). Influence of mathematics anxiety on mathematics performance: Mediating effects of mathematical engagement. Mathematics Education Research Journal (first published online). 10.1007/s13394-025-00536-1

[bjep70057-bib-0111] Pekrun, R. , Lichtenfeld, S. , Marsh, H. W. , Murayama, K. , & Goetz, T. (2017). Achievement emotions and academic performance: Longitudinal models of reciprocal effects. Child Development, 88(5), 1653–1670. 10.1111/cdev.12704 28176309

[bjep70057-bib-0112] Petronzi, D. , Staples, P. , Sheffield, D. , Hunt, T. , & Fitton‐Wilde, S. (2019). Further development of the Children's mathematics anxiety scale UK (CMAS‐UK) for ages 4–7 years. Educational Studies in Mathematics, 100, 231–249. 10.1007/s10649-018-9860-1

[bjep70057-bib-0113] Pletzer, B. , Wood, G. , Moeller, K. , Nuerk, H. C. , & Kerschbaum, H. H. (2010). Predictors of performance in a real‐life statistics examination depend on the individual cortisol profile. Biological Psychology, 85(3), 410–416. 10.1016/j.biopsycho.2010.08.015 20837092

[bjep70057-bib-0114] Putwain, D. , & Daly, A. L. (2014). Test anxiety prevalence and gender differences in a sample of English secondary school students. Educational Studies, 40(5), 554–570. 10.1080/03055698.2014.953914

[bjep70057-bib-0115] Ramirez, G. , & Beilock, S. L. (2011). Writing about testing worries boosts exam performance in the classroom. Science, 331, 211–213. 10.1126/science.119942 21233387

[bjep70057-bib-0116] Ramirez, G. , Gunderson, E. A. , Levine, S. C. , & Beilock, S. L. (2013). Math anxiety, working memory, and math achievement in early elementary school. Journal of Cognition and Development, 14, 187–202. 10.1080/15248372.2012.664593

[bjep70057-bib-0117] Richardson, F. , & Suinn, R. (1972). The mathematics anxiety rating scale: Psychometric data. Journal of Counseling Psychology, 19, 551–554. 10.1037/h0033456

[bjep70057-bib-0118] Rossi, S. , Xenidou‐Dervou, I. , & Cipora, K. (2023). Emotions and mathematics: Anxiety profiles and their influence on arithmetic performance in university students. Royal Society Open Science, 10, 230861. 10.1098/rsos.230861 37830022 PMC10565394

[bjep70057-bib-0119] Rossi, S. , Xenidou‐Dervou, I. , Simsek, E. , Artemenko, C. , Daroczy, G. , Nuerk, H. , & Cipora, K. (2022). Mathematics–gender stereotype endorsement influences mathematics anxiety, self‐concept, and performance differently in men and women. Annals of the New York Academy of Sciences, 1513(1), 113–121. 10.1111/nyas.14779 PMC9545177 35429357

[bjep70057-bib-0120] Sarfo, J. O. , Garcia‐Santillan, A. , Adusei, H. , Mokhanova, V. S. , Drushlvak, M. , Semenikhina, O. , Donveh, A. P. S. , Zand, F. S. , Najafi, R. , Enea, V. , Malik, S. , Ashrafi, F. , Malik, N., I , Ansah, E. W. , Wongcharee, H. , Egara, F. O. , Tipandjan, A. , Cudjoe, J. , Azam, U. , … Vally, Z. (2020). Gender differences in mathematics anxiety across cultures: A univariate analysis of variance among samples from twelve countries. European Journal of Contemporary Education, 9, 878–885. 10.1037/a0034099

[bjep70057-bib-0121] Sarı, M. H. , & Hunt, T. E. (2020). Parent‐child mathematics affect as predictors of children's mathematics achievement. International Online Journal of Primary Education, 9(1), 85–96.

[bjep70057-bib-0122] Sarkar, A. , Dowker, A. , & Cohen Kadosh, R. (2014). Cognitive enhancement or cognitive cost: Trait‐specific outcomes of brain stimulation in the case of mathematics anxiety. Journal of Neuroscience, 34, 16605–16610. 10.1523/JNEUROSCI.3129-14.2014 25505313 PMC4261089

[bjep70057-bib-0123] Sidney, P. G. , Scheibe, D. A. , Zahrn, L. , Brown, K. G. I. , & Thompson, C. A. (2024). Developing effective interventions for math anxiety. Current Directions in Psychological Science, 34(1), 57–63. 10.1177/09637214241300111

[bjep70057-bib-0125] Simmons, F. R. , Soto‐Calvo, E. , Adams, A. M. , Francis, H. , Patel, H. , & Hartley, C. (2024). Longitudinal associations between parental mathematics anxiety and attitudes and young children's mathematics attainment. Journal of Experimental Child Psychology, 238, 105779. 10.1016/j.jecp.2023.105779 37783015

[bjep70057-bib-0126] Skagerlund, K. , Ostergren, R. , Vastfjall, D. , & Traff, U. (2019). How does mathematics¨ anxiety impair mathematical abilities? Investigating the link between math anxiety, working memory, and number processing. PLoS One, 14(1), e0211283. 10.1371/journal.pone.0211283 30682125 PMC6347150

[bjep70057-bib-0127] Soni, A. , & Kumari, S. (2017). The role of parental math anxiety and math attitude in their children's math achievement. International Journal of Science and Mathematics Education, 15(2), 331–347. 10.1007/s10763-015-9687-5

[bjep70057-bib-0128] Sorvo, R. , Koponen, T. , Viholainen, H. , Aro, T. , Raikkonen, E. , Peura, P. , Dowker, A. , & Aro, M. (2017). Math anxiety and its relationship with basic arithmetic skills among primary school children. British Journal of Educational Psychology, 87, 309–327. 10.1111/bjep.12151 28258597

[bjep70057-bib-0129] Spelke, E. S. (2005). Sex differences in intrinsic aptitude for mathematics and science?: A critical review. American Psychologist, 60(9), 950–958. 10.1037/0003-066X.60.9.950 16366817

[bjep70057-bib-0130] Spencer, S. J. , Steele, C. M. , & Quinn, D. M. (1999). Stereotype threat and women's math performance. Journal of Experimental Social Psychology, 35(1), 4–28. 10.1006/jesp.1998.1373

[bjep70057-bib-0131] Spielberger, C. D. (1972). Anxiety: Current trends in theory and research. Academic Press. 10.1016/B978-0-12-657401-2.50008-3

[bjep70057-bib-0133] Sumpter, L. , & Sollerman, S. (2023). Changes in affect: Patterns in grade 4 and grade 8 students' expressed emotional directions. International Journal of Mathematical Education in Science and Technology, 54(8), 1598–1613. 10.1080/0020739X.2023.2184279

[bjep70057-bib-0134] Supekar, K. , Iuculano, T. , Chen, L. , & Menon, V. (2015). Remediation of childhood math anxiety and associated neural circuits through cognitive tutoring. Journal of Neuroscience, 35, 12574–12583. 10.1523/jneurosci.0786-15.2015 26354922 PMC4563039

[bjep70057-bib-0135] Svraka, B. , Álvarez, C. , & Szücs, D. (2024). Anxiety predicts math achievement in kindergarten children. Frontiers in Psychology, 15, 1335952. 10.3389/fpsyg.2024.1335952 38476390 PMC10927750

[bjep70057-bib-0136] Szczygiel, M. (2020a). Gender, general anxiety, math anxiety and math achievement in early school‐age children. Issues in Educational. Research, 30, 1126–1142. www.iier.org.au/iier30/szczygiel.pdf

[bjep70057-bib-0137] Szczygiel, M. (2020b). When does math anxiety in parents and teachers predict math anxiety and math achievement in elementary school children? The role of gender and grade year. Social Psychology of Education, 23, 1023–1054. 10.1007/s11218-020-09570-2

[bjep70057-bib-0138] Szczygieł, M. , & Pieronkiewicz, B. (2021). Exploring the nature of math anxiety in young children: Intensity, prevalence, reasons. Mathematical Thinking and Learning, 24, 1–19. 10.1080/10986065.2021.1882363

[bjep70057-bib-0139] Szczygieł, M. , Szucs, D. , & Toffalini, E. (2024). Math anxiety and math achievement in primary school children: Longitudinal relationship and predictors. Learning and Instruction, 92, 101906. 10.1016/j.learninstruc.2024.101906

[bjep70057-bib-0140] Szucs, D. , & Mammarella, I. C. (2024). A biopsychological–social view of mathematical development. Current Opinion in Behavioral Sciences, 55, 101332. 10.1016/j.cobeha.2023.101332

[bjep70057-bib-0142] Thomas, G. , & Dowker, A. (2000). Mathematics anxiety and related factors in young children . Paper presented at British Psychological Society Developmental Section Conference, Bristol, UK, September 2000.

[bjep70057-bib-0143] Thomson, S. , Wernert, N. , Rodrigues, S. , & O'Grady, E. (2020). TIMSS 2019 Australia. Volume I: Student performance. Australian Council for Education Research Ltd.

[bjep70057-bib-0144] Tomasetto, C. , Mirisola, A. , Galdi, S. , & Cadinu, M. (2015). Parents' math‐gender stereotypes, children's self‐perception of ability, and children's appraisal of parents' evaluations in 6‐year‐olds. Contemporary Educational Psychology, 42, 186–198. 10.1016/j.cedpsych.2015.06.007

[bjep70057-bib-0145] Van der Beek, J. P. J. , Van der Ven, S. H. G. , Kroesbergen, E. H. , & Leseman, P. P. M. (2017). Self‐concept mediates the relation between achievement and emotions in mathematics. British Journal of Educational Psychology, 87(3), 478–495. 10.1111/bjep.12160 28440010

[bjep70057-bib-0146] Van Mier, H. , Schleepen, T. , & den Van Berg, F. (2018). Gender differences regarding the impact of math anxiety on arithmetic performance in second and fourth graders. Frontiers in Psychology, 9, 2690. 10.3389/fpsyg.2018.02690 30713516 PMC6345718

[bjep70057-bib-0147] Vanbinst, K. , Bellon, E. , & Dowker, A. (2020). Mathematics anxiety: An intergenerational approach. Frontiers in Psychology, 11, 1648. 10.3389/fpsyg.2020.01648 32793047 PMC7385133

[bjep70057-bib-0148] Vukovic, R. K. , Kieffer, M. J. , Bailey, S. P. , & Harari, R. R. (2013). Mathematics anxiety in young children: Concurrent and longitudinal associations with mathematical performance. Contemporary Educational Psychology, 38(1), 1–10. 10.1016/j.cedpsych.2012.09.001

[bjep70057-bib-0149] Wang, Z. , Hart, S. A. , Kovas, Y. , Lukowski, S. , Soden, B. , Thompson, L. A. , & Petrill, S. (2014). Who is afraid of math? Two sources of genetic variance for mathematical anxiety. Journal of Child Psychology and Psychiatry, 55(9), 1056–1064. 10.1111/jcpp.12224 24611799 PMC4636726

[bjep70057-bib-0150] Wang, Z. , Lukowski, S. L. , Hart, S. A. , Lyons, I. M. , Thompson, L. A. , Kovas, Y. , Mazzocco, M. M. M. , Plomin, R. , & Petrill, S. A. (2015). Is math anxiety always bad for math learning? The role of math motivation. Psychological Science, 26(12), 1863–1876. 10.1177/0956797615602471 26518438 PMC4679544

[bjep70057-bib-0151] Wang, Z. , Oh, W. , Malanchini, M. , & Borriello, G. A. (2020). The developmental trajectories of mathematics anxiety: Cognitive, personality, and environmental correlates. Contemporary Educational Psychology, 61, 101876. 10.1016/j.cedpsych.2020.101876

[bjep70057-bib-0152] Wang, Z. , Shakeshaft, N. , Schofield, K. , & Malanchini, M. (2018). Anxiety is not enough to drive me away: A latent profile analysis on math anxiety and math motivation. PLoS One, 13, e0192072. 10.1371/journal.pone.019207 29444137 PMC5812593

[bjep70057-bib-0153] Wei, W. , Xu, C. , Caviola, S. , & Mammarella, I. C. (2024). Affective and cognitive factors associated with Chinese and Italian children's arithmetic performance. BMC Psychology, 12, 466. 10.1186/s40359-024-01965-6 39217405 PMC11366164

[bjep70057-bib-0154] Wigfield, A. , & Meece, J. L. (1988). Math anxiety in elementary and secondary school students. Journal of Educational Psychology, 80, 210–216. 10.1037/0022-0663.80.2.210

[bjep70057-bib-0155] Williams, K. , White, S. , & English, L. (2024). Profiles of general, test, and mathematics anxiety in 9‐ and 12‐year‐olds: Relations to gender and mathematics achievement. Mathematics Education Research Journal, 37, 161–186. 10.1007/s13394-024-00485-1

[bjep70057-bib-0156] Wood, G. , Pinheiro‐Chagas, P. , Júlio‐Costa, A. , Micheli, L. R. , Krinzinger, H. , Kaufmann, L. , Willmes, K. , & Haase, V. (2012). Math anxiety questionnaire: Similar latent structure in Brazilian and German school children. Child Development Research, 2012, 610192. 10.1155/2012/610192

[bjep70057-bib-0157] Wu, S. S. , Barth, M. , Amin, H. , Malcarne, V. , & Menon, V. (2012). Math anxiety in second and third graders and its relation to mathematics achievement. Frontiers in Psychology, 3, 162. 10.3389/fpsyg.2012.00162 22701105 PMC3369194

[bjep70057-bib-0158] Yu, X. , Zhou, H. , Sheng, P. , Ren, B. , Wang, Y. , Wang, H. , & Zhou, X. (2023). Math anxiety is more closely associated with math performance in female students than in male students. Current Psychology, 43, 1381–1394. 10.1007/s12144-023-04349-y

[bjep70057-bib-0160] Zhang, J. , Zhao, N. , & Kong, Q. P. (2019). The relationship between math anxiety and math performance: A meta‐analytic investigation. Frontiers in Psychology, 10, 1613. 10.3389/fpsyg2019 31447719 PMC6692457

[bjep70057-bib-0161] Park, D. R. , Ramirez, G. , & Beiock, S. L. (2014). The role of expressive writing in math anxiety. Journal of Experimental Psychology, 20, 103–111. 10.1037/xap0000013 24708352

[bjep70057-bib-0162] Gierl, M. J. , & Busanz, J. (1995). Anxieties and attitudes related to mathematics in grades 3 and 6. The Journal of Experimental Education, 63, 138–158. 10.1080/00220973.1995.99443818

[bjep70057-bib-0163] Newstead, K. (1998). Aspects of children's mathematics anxiety. Educational Studies in Mathematics, 36, 53–71. 10.1023/A:1003177809664

[bjep70057-bib-0164] Suinn, R. M. , Taylor, S. , & Edwards, R. W. (1988). Suinn Mathematics Anxiety Rating Scale for elementary school students (MARS=E): Psychommetric and normative data. Educational and Psychological Measurement, 48, 979–994. 10.1177/001316448848013

[bjep70057-bib-0165] Yuan, Z. , Tan, T. , & Ye, R. (2023). A cross‐national study of mathematics anxiety. The Asia‐Pacific Education Research, 32, 295–306. 10.1007/s40299-022-00652-7

[bjep70057-bib-0166] Carraher, T. N. , Carraher, D. W. , & Schliemann, A. D. (1985). Mathematics in the streets and in schools. British Journal of Developmental Psychology, 3, 21–29. 10.1111/j2044-835x.1985.tb100951.x

[bjep70057-bib-0167] Dowker, A. (2015). Individual differences in arithmetical abilities: The componential nature of arithmetic. In R. Cohen Cadosh & A. Dowker (Eds.), Oxford handbook of numerical cognition (pp. 878–894). Oxford University Press.

[bjep70057-bib-0168] Dowker, A. (2023). The componential nature of arithmetical cognition: Some important questions. Frontiers in Psychology, 14, 11882771. 10.3380/fpsyg2023.11882771 PMC10536274 37780151

